# Development of selective bispecific Wnt mimetics for bone loss and repair

**DOI:** 10.1038/s41467-021-23374-8

**Published:** 2021-05-31

**Authors:** Tristan W. Fowler, Troy L. Mitchell, Claudia Y. Janda, Liqin Xie, Shengjiang Tu, Hui Chen, Haili Zhang, Jingjing Ye, Brian Ouyang, Tom Z. Yuan, Sung-Jin Lee, Maureen Newman, Nikita Tripuraneni, Erica S. Rego, Devin Mutha, Archana Dilip, Meghah Vuppalapaty, Helene Baribault, Wen-Chen Yeh, Yang Li

**Affiliations:** 1 Surrozen, Inc., South San Francisco, CA USA; 2 Present Address: Princess Maxima Center for Pediatric Oncology, Utrecht, The Netherlands

**Keywords:** Antibody therapy, Regenerative medicine, Skeleton

## Abstract

The Wnt signaling pathway is intricately connected with bone mass regulation in humans and rodent models. We designed an antibody-based platform that generates potent and selective Wnt mimetics. Using this platform, we engineer bi-specific Wnt mimetics that target Frizzled and low-density lipoprotein receptor-related proteins and evaluate their effects on bone accrual in murine models. These synthetic Wnt agonists induce rapid and robust bone building effects, and correct bone mass deficiency and bone defects in various disease models, including osteoporosis, aging, and long bone fracture. Furthermore, when these Wnt agonists are combined with antiresorptive bisphosphonates or anti-sclerostin antibody therapies, additional bone accrual/maintenance effects are observed compared to monotherapy, which could benefit individuals with severe and/or acute bone-building deficiencies. Our data support the continued development of Wnt mimetics for the treatment of diseases of low bone mineral density, including osteoporosis.

## Abstract (web summary)

Antibody-based Wnt agonists are able to phenocopy Wnt signaling in vivo resulting in increased bone density, repair, and strength. Here, the authors show that Wnt agonists can reverse bone loss associated with ovariectomy and build stronger bone when administered after fracture.

## Introduction

Diseases of low bone mineral density (BMD), including osteoporosis, present serious global health concerns for both men and women. Osteoporosis-related fragility fractures are associated with significant morbidity and mortality^1–5^. It has been estimated that the lifetime risk for osteoporotic fracture at age 50 is ~20% for men and 50% for women^6^. Using Fracture Risk Assessment Tool (FRAX) models, 158 million people (21 million men and 137 million women) over age 50 years were estimated to be at high risk for an osteoporotic fracture worldwide in 2010, with an expected doubling of this number by 2040^7^. Antiresorptive agents and bone anabolic drugs are the mainstays of therapy for osteoporosis, but different therapies are still needed to address the clinical unmet needs and provide alternative therapeutic options^8,9^. In addition, other conditions of low bone mineral density, including osteogenesis imperfecta, renal osteodystrophy, and disuse osteopenia have proven difficult to treat and therapies are needed to overcome this treatment gap^10–12^.

Wnt (“Wingless-related integration site” or “Wingless and Int-1” or “Wingless-Int”) ligands and their signaling activators play key roles in controlling the development, homeostasis, and regeneration of many essential organs and tissues, including bone^13–15^. Specifically, osteoblasts, osteocytes, and osteoclasts (the three major cell types of bone), all have been shown to be regulated by Wnt/β-catenin-dependent signaling^16–19^. This has been observed along the entire osteoblast lineage, including in osteocytes where modulation of sclerostin is required in response to mechanical load-induced bone accrual^20,21^. Additionally, Wnt signaling has been shown to reduce the ratio of receptor activator of nuclear factor-κB ligand (RANKL)/osteoprotegerin (OPG) expression, which indirectly reduces osteoclast activation^19^.

Direct activation of Wnt signaling has the potential to enhance bone accrual and repress bone resorption in disease settings. One of the challenges in engineering a therapeutic that directly activates Wnt signaling is the existence of 19 Wnt ligands and multiple possible combinations of Wnts with receptors, including Frizzled 1–10 (Fzd_1–__10_) and low-density lipoprotein receptor-related protein 5 and 6 (Lrp5, Lrp6). In addition, Wnt proteins are highly modified post-translationally. Palmitoleoylation on a conserved serine residue is essential for Wnt interaction and signaling through their receptors^22^. Partly due to this post-translational palmitoleoylation, Wnts are hydrophobic, making them difficult to express and purify. The anti-Fzd antibody 18R5 is a Fzd_1,2,5,7,8_ binder^23^, and the Lrp binder DKK1c binds to both Lrp5 and 6^24,25^. To resolve these challenges associated with Wnts, a Wnt mimetic linking the Fzd binder (18R5 in a single-chain variable fragment [scFv] format) and the Lrp binder (DKK1c) into a single polypeptide chain (18R5-DKK1c) has been previously shown to have the potential to activate Wnt/β-catenin signaling in various tissue systems, including liver, bone, and intestine^23,26^. However, the functional impact of this molecule in in vivo systems remained to be clearly demonstrated. We have also recently developed a platform for potent and selective Wnt mimetic generation and engineered water soluble and easily manufacturable IgG-based bi-specific molecules that bind to specific Fzd(s) and Lrp5 or 6^27^. We identified multimerization of Fzd and Lrp, with optimal stoichiometry of two Fzds and one or two Lrps, as a requirement for maximal Wnt/β-catenin activation^27^.

In the studies presented here, we first examined whether 18R5-DKK1c can be delivered systemically to induce bone accrual in vivo. To test this hypothesis, we delivered 18R5-DKK1c through adeno-associated virus (AAV) in a mouse model and measured longitudinal changes in bone accrual using a variety of analyses. In parallel, we engineered tetravalent bi-specific antibody-based molecules with different Fzd and Lrp binders and specificities. We then examined the efficacy of these recombinant Wnt mimetic proteins on bone building in various disease models, including osteoporosis, aging, and long bone fracture. We found that these Wnt mimetics had robust bone-building effects, thus supporting their continued development as therapeutic antibodies to modulate the Wnt pathway for tissue regeneration.

## Results

### Wnt receptor expression profiles from bone cells

**Fig. 1:**
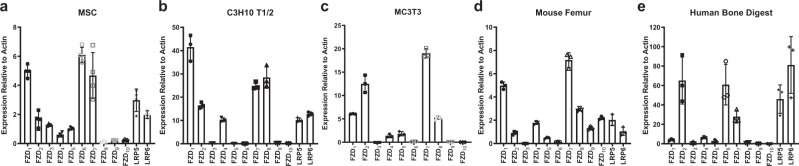
Wnt signaling components expression analysis from various cells and species. Fzd_1–__10_, along with Lrp5, and Lrp6 mRNA expression in **a** mesenchymal stem cells (MSC), **b** C3H10T1/2 cells, **c** MC3T3 cells, **d** mouse femur, and **e** human cancellous femoral head quantified by qPCR and normalized by the housekeeping gene Actin. Bars represent means ± SD. **d** *n* = 3 mice and **e** *n* = 3 human femoral heads.

To guide the selection of Wnt mimetics with the appropriate Fzd specificity to stimulate bone accrual, we investigated Fzd and Lrp expression on osteoblasts or precursors of osteoblasts. We first examined mesenchymal stem cells (MSCs) and found that they expressed a wide range of Fzd receptors, including expression of Fzd_1,6,7_ and Fzd_2,3,4,5_, as well as both Lrp5 and 6 (Fig. 1a), suggesting that these multipotential precursors could be stimulated through a variety of Fzd and Lrp specific binder combinations. Next, we studied C3H10T1/2, a fibroblast cell line with MSC-like characteristics that can be differentiated into multiple lineages, including osteoblasts and chondroblasts^28^. The Fzd expression profile showed expression of Fzd_1,2,4,7,8_ (Fig. 1b) and both Lrp5 and 6, suggesting that C3H10T1/2 cells may be stimulated by these Fzd and Lrp binder combinations. To understand expression of Wnt receptors on cells committed toward the osteoblast lineage, we examined receptor expression on an osteoblast cell line, MC3T3 cells. The expression analysis showed that these cells had possible expression of Fzd_1,2,7,8_ and Fzd_4,5_ (Fig. 1c). Examination of Fzd and Lrp expression in mouse (Fig. 1d) and human (Fig. 1e) femoral bones, primary bone tissues that contain multiple cell types, showed expression of Fzd_1_ or Fzd_2_, and Fzd_6,7_ as well as both Lrp5 and Lrp6.

### AAV-delivered Wnt-mimetic 18R5-DKK1c induces bone accrual

We previously found that the Wnt mimetic 18R5-DKK1c (with the specificity for the Fzd_1,2,7_ and Fzd_5,8_ subfamilies) increased alkaline phosphatase (ALP) expression in vitro in cells of osteoblast lineage in the absence and presence of bone morphogenetic protein 2^26^. Thus, we next investigated whether 18R5-DKK1c was capable of stimulating bone accrual in vivo.

We first evaluated the achievability of delivering 18R5-DKK1c systemically using an AAV-mediated expression of 18R5-DKK1c in a murine model. On Day 0, mice received a single tail vein injection of either AAV containing vectors encoding fusion proteins or control genes, and then were followed for 28 days. For negative controls, we used vehicle (phosphate-buffered solution (PBS), AAV expressing anti-green fluorescent protein (GFP), and AAV expressing a fusion protein of anti-GFP scFv fused to mutated DKK1c incapable of binding to Lrp5/6 (scFv(anti-GFP)-DKK1cF234K). These negative controls were utilized to ensure that the effects of 18R5-DKK1c were not influenced by either the use of AAV or the format and fusion of the scFv protein. For a positive control, we utilized anti-sclerostin antibody protein at 10 mg/kg, administered subcutaneously twice weekly for 4 weeks (total eight doses). Blood samples were drawn on days 7, 14, 21, and 28 of the study period. Both 18R5-DKK1c and scFv(anti-GFP)-DKK1cF234K were Flag-tagged and detected in the serum by anti-Flag antibody via ELISA testing, confirming systemic delivery was attained (Supplementary Fig. 1a). There were no measurable changes in body weight (Supplementary Fig. 1b) or whole-body fat percentage (Supplementary Fig. 1c), as measured by dual energy x-ray absorptiometry (DEXA), comparing day 0 to day 28. However, there was an approximate 20% increase in liver weight when normalized to total body weight in the 18R5-DKK1c group compared to vehicle (Supplementary Fig. 1d). Colon and small intestine length and weight were unchanged (Supplementary Fig. 1e–h), and liver, colon, and small intestine histopathology was within normal range (Supplementary Fig. 1i). Small intestine and liver sections stained with proliferation marker, Ki67, revealed no differences across groups (Supplementary Fig. 1j).

**Fig. 2:**
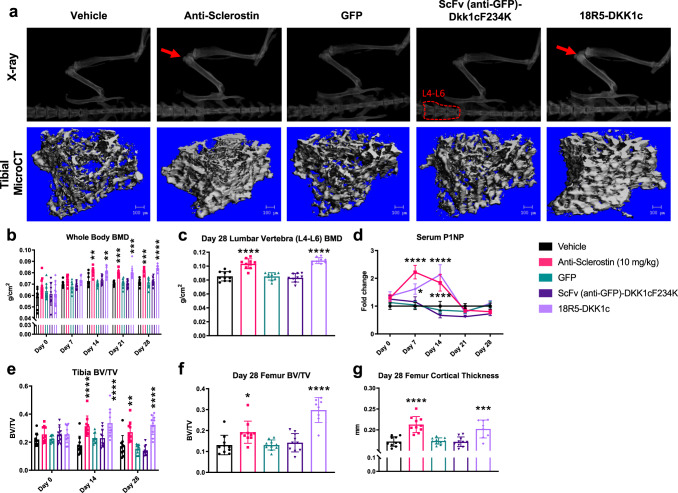
Radiographic and serum analyses of bone accrual induced by Wnt mimetics. **a** Representative x-ray (top row) and micro-computed tomography (micro-CT) (bottom row) images at day 28. For x-ray, individual sample regions of interest (ROI) quantified the longitudinal (**b**) average whole-body bone mineral density (BMD). **a** Red arrows point to increased bone mineral (radiographic contrast) in the distal femur and proximal tibia. Red dotted regions demarcate the ROI analyzed for **c** average lumbar vertebra (L4–L6). **d** Serum samples obtained at days 0, 7, 14, 21, and 28 were assessed for procollagen type-1 N-terminal propeptide (P1NP). For each time point, the serum P1NP levels for all test groups are normalized to the average of vehicle-treated P1NP serum concentration. **e** Longitudinal average tibial BV/TV at days 0, 14, and 28. **f** Day 28 ex vivo endpoint micro-CT quantification of the distal femoral epiphyseal BV/TV and **g** mid-shaft cortical thickness collected at termination. Statistical significance was determined by two-way ANOVA for (**b**, **d**, **e**) and one-way ANOVA for all others. Graphs represent mean values ± SD, wherein *p < 0.05, **p < 0.01, ***p < 0.001, ****p < 0.0001. Statistical analyses where the following letters define groups (a = Vehicle, b = Anti-Sclerostin, c = 18R5-Dkk1c). **b** Day 14, a vs. b, *p* = 0.0049; a vs. c, *p* = 0.0031; Day 21, a vs. b, *p* = 0.0005; a vs. c, *p* = 0.0002; Day 28: a vs. b, *p* = 0.0006; a vs. c, *p* ≤ 0.0001. **c** a vs. b, *p* ≤ 0.0001; a vs. c, *p* ≤ 0.0001. **d** Day 7, a vs. b, *p* ≤ 0.0001; a vs. c, *p* = 0.017; Day 14, a vs. b, *p* ≤ 0.0001, a vs. c, *p* ≤ 0.0001. **e** Day 14, a vs. b, *p* ≤ 0.0001; a vs. c, *p* ≤ 0.0001; Day 28, a vs. b, *p* = 0.0033; a vs. c, *p* ≤ 0.0001. **f** a vs. b, *p* = 0.0388; a vs. c, *p* ≤ 0.0001. **g** a vs. b, *p* ≤ 0.0001; a vs. c, *p* = 0.0009. **b**–**g** *n* = 10 mice/group.

We next examined the potential role of 18R5-DKK1c in stimulating bone accrual. Compared to vehicle, systemic expression of 18R5-DKK1c significantly increased whole-body BMD as early as day 14 and BMD remained increased at day 28, as determined by DEXA (Fig. 2a, b). The increase in BMD with 18R5-DKK1c was similar to that observed for anti-sclerostin antibody. We also observed an increase in BMD in lumbar vertebrae L4–L6 (Fig. 2c), which was measured at day 28 only. Transient significant increases from day 0 in procollagen-1-N-terminal peptide (P1NP), a marker of bone formation, were observed with anti-sclerostin antibody treatment and 18R5-DKK1c (Fig. 2d). Compared to vehicle, 18R5-DKK1c significantly increased bone volume/total volume (BV/TV) in the proximal tibia, as assessed longitudinally over the 28-day study period via micro-CT analysis (Fig. 2e). In addition, femurs measured at day 28 showed significantly increased BV/TV at the distal epiphysis (Fig. 2f) and cortical thickness of the mid-diaphysis (Fig. 2g) with 18R5-DKK1c treatment (Supplementary Table 2). The BMD and BV/TV increases observed at day 14 were sustained through day 28.

**Fig. 3:**
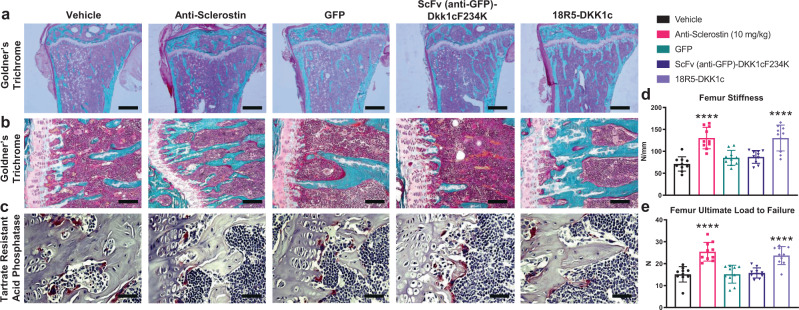
Histology and mechanical testing of bone accrual induced by Wnt mimetics. **a**, **b** Tibias were isolated after 28 days, formalin fixed, embedded in methyl methacrylate, sectioned longitudinally, and stained. Representative images of tibial sections from each treatment group at **a** 4× scale bar 1 mm and **b** 10× scale bar 200 µm stained with Goldner’s trichrome (mineralized bone is colored blue) and **c** 40× scale bar 50 µm stained for tartrate resistant acid phosphatase (TRAP). Femurs collected at study termination (day 28) were analyzed for bone strength at the mid-shaft femur by a standard 3-point bending test and quantified for **d** average femur stiffness and **e** average ultimate load to failure. **a**–**c** Representative images from *n* = 10 samples/group having similar results. **d**, **e** Bar graphs represent mean values ± SD. Statistical significance was determined by one-way ANOVA, where ****p < 0.0001. The following letters define groups (a = Vehicle, b = Anti-Sclerostin, c = 18R5-DKK1c). **d** a vs. b, *p* ≤ 0.0001; a vs. c, *p* ≤ 0.0001. **e** a vs. b, *p* ≤ 0.0001; a vs. c, *p* ≤ 0.0001. *n* = 10 mice/group.

To further examine the underlying changes in bone histology and cell physiology, histomorphometric analyses of proximal tibia were conducted at the end of the 28-day study period. We found that treatment with 18R5-DKK1c compared to vehicle resulted in significantly increased trabecular number, trabecular thickness, and osteoid thickness (Supplementary Table 1), consistent with an increase of bone volume percentage. Furthermore, 18R5-DKK1c compared to vehicle significantly increased osteoblast surface, suggesting an anabolic mechanism of action (Fig. 3a–c, Supplementary Table 1).

To ensure the increased bone mass induced by 18R5-DKK1c was physiological and functional, we conducted biomechanical strength testing on bones collected at study termination (day 28). 18R5-DKK1c treatment increased both bone stiffness (Fig. 3d) and ultimate load to fracture (Fig. 3e) in femurs when tested by 3-point bending, demonstrating improved resistance to fracture. The positive effects on bone strength with 18R5-DKK1c were similar to the effects observed with anti-sclerostin antibody and significantly higher than vehicle control.

### Generation of Wnt mimetics

**Fig. 4:**
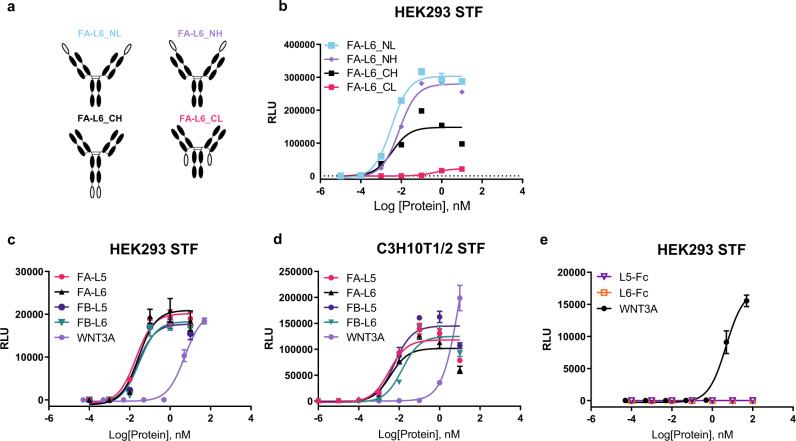
In vitro characterization of Wnt mimetic molecules. **a** Diagram of the tetravalent bi-specific Wnt mimetic formats. VHH domain is represented by the open oval symbol and IgG by the black-filled oval symbol. **b**–**e** Wnt signaling STF activity is represented by relative luminescence units (RLU). **b** The dose-dependent STF activities of the four different VHH-IgG fusion mimetics shown in **a**. **c** Dose-dependent STF activities of the FA-L5, FA-L6, FB-L5, FB-L6, and WNT3A in HEK293 cells. **d** Dose-dependent STF activities of FA-L5, FA-L6, FB-L5, FB-L6, and WNT3A in C3H10T1/2 cells. **e** Dose-dependent STF activities of L5-Fc, L6-Fc, and WNT3A in HEK293 cells. Data are representative of three independent experiments performed in triplicates and are shown as mean ± SD.

Although 18R5-DKK1c provided in vivo proof-of-concept for a Wnt mimetic to stimulate bone accrual, it was not suited to further pursue as a potential therapeutic molecule due in part to the poor biophysical properties of the DKK1c component of the molecule which resulted in low expression and the appearance of multiple protein species of the fusion molecule. Thus, we generated another Wnt mimetic with specificity for Fzd_1,2,5,7,8_, similar to 18R5-DKK1c. In addition, since the Fzd_1,2,7_ subfamily was expressed in all osteoblast or osteoblast precursor cells we profiled (Fig. 1a–c), we tested whether narrowing Fzd specificity to the Fzd_1,2,7_ subfamily would be sufficient to stimulate bone accrual. Therefore, for Fzd binders we selected two proprietary Fzd binding IgGs. The first one binds to Fzd_1,2,5,7,8_, referred to herein as FA, and the second one binds to Fzd_1,2,7_, referred to as FB (Supplementary Fig. 1a–d). For Lrp binders, we selected two proprietary single domain antibodies (VHH) derived from a camelid phage library: an Lrp6 biased binder, referred herein as L6 and an Lrp5 biased binder, referred herein as L5 (Supplementary Fig. 2e, f). These four binders are distinct from the binders described previously^27^ but are assembled into tetravalent bispecific formats as shown in Fig. 4a, the stoichiometry identified previously to be one of the most efficient format for Wnt signal activation^27^. Since the Lrp binding VHH domain can be attached to four different locations on the Fzd binding IgG, we first tested the four different combinations between L6 and FA (depicted in Fig. 4a). While all four combinations activated Wnt signaling as detected in Wnt responsive HEK293 Super TopFlash (STF) reporter cells, the attachment of VHH to the N-terminal end of either the heavy (NH) or light (NL) chain resulted in higher activity than attachment to the C-terminal ends (Fig. 4b), with half-maximal effective concentration (EC_50_) values in the pM range. Based on these results, fusion of the VHH domain to the N terminus of light chain (NL) was selected for further evaluation in the studies described herein. As shown in Fig. 4c, the four different combinations of the Fzd and Lrp binders, FA-L5, FA-L6, FB-L5, and FB-L6 fusions of VHH domain to the N terminus of light chain are strongly efficacious in inducing Wnt signaling compared to recombinant human WNT3A in HEK293 cells. Additionally, these four Wnt mimetics induced Wnt signaling in the osteoblast precursor cell line, C3H10T1/2 cells, by several orders of magnitude greater potency when compared to WNT3A (Fig. 4d). To assess the effects of L5 and L6 binding arms alone on Wnt signaling, they were fused to a Fc, instead of to anti-Fzd antibodies to generate L5-Fc and L6-Fc. As shown in Fig. 4e, in the absence of Fzd binding arms, Lrp binders alone (as the bivalent Fc fusion molecules) were not able to induce Wnt signal.

### Wnt mimetics stimulate bone accrual

**Fig. 5:**
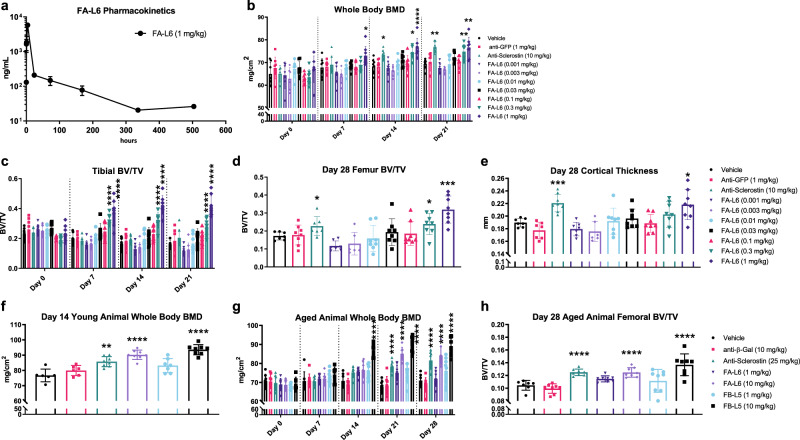
Wnt mimetic antibodies robustly stimulate bone accrual in naive young and older mice. **a** Serum concentrations of FA-L6 antibody after intraperitoneal administration, as measured by ELISA over a 21-day period. Each time point represents the average assessed in three animals, with error bars representing the standard deviation of the mean. **b**–**e** 8-week-old C57BL6/J female mice were injected bi-weekly for 28 days intraperitoneally with increasing concentrations of FA-L6 antibody and compared to a negative control antibody (anti-GFP IgG 1 mg/kg), and a positive control (bi-weekly subcutaneous administration of 10 mg/kg anti-sclerostin antibody). **b** Longitudinal DEXA measurements of whole-body BMD quantified for days 0, 7, 14, and 21. **c** Longitudinal micro-CT of proximal tibial bone volume/tissue volume (BV/TV) quantified on days 0, 7, 14, and 21. **d** Endpoint distal femoral BV/TV and **e** mid-shaft cortical thickness were quantified at day 28. **f** Whole-body BMD by DEXA in 16-week-old C57BL6/J female mice injected bi-weekly for 14 days with FA-L6 (10 mg/kg) or FB-L5 (1 or 10 mg/kg) antibody, or a negative control antibody (anti-β-gal 10 mg/kg), or positive control (anti-sclerostin antibody 25 mg/kg). **g**, **h** 1-year-old C57BL6/J female mice were injected bi-weekly for 28 days intraperitoneally with 1 and 10 mg/kg of FA-L6 or FB-L5 antibody or a negative control antibody (anti-β-gal) or positive control (bi-weekly subcutaneous administration of 25 mg/kg anti-sclerostin antibody). **g** Longitudinal whole-body BMD over time and **h** day 28 endpoint distal femoral BV/TV measurements are shown. **a** Graph represents mean ± SD, *n* = 3 mice. **b**–**h** Bar graphs represent mean values ± SD. Statistical significance was determined by two-way ANOVA for **b**, **c**, **g** and one-way ANOVA for all others (*p < 0.05, **p < 0.01, ***p < 0.001, ****p < 0.0001) where the following letters define groups (a = Vehicle, b = Anti-Sclerostin, c = FA-L6 (0.3 mg/kg), d = FA-L6 (1 mg/kg), e = FA-L6 (10 mg/kg), f = FB-L5 (10 mg/kg)). **b** Day 7, a vs. d, *p* = 0.041; Day 14, a vs. b, *p* = 0.032, a vs. c, *p* = 0.0412, a vs. d, *p* ≤ 0.0001; Day 21, a vs. b, *p* = 0.0019, a vs. c, *p* = 0.0092, a vs. d, *p* = 0.0024. **c** Day 7, a vs. c, *p* ≤ 0.0001; a vs. d, *p* ≤ 0.0001. Day 14, a vs. c, *p* ≤ 0.0001; a vs. d, *p* ≤ 0.0001. Day 21, a vs. c, *p* ≤ 0.0001; a vs. d, *p* ≤ 0.0001. **d** a vs. b, *p* = 0.0183, a vs. c, *p* = 0.0141, a vs. d, *p* = 0.0002. **e** a vs. b, *p* = 0.001, a vs. d, *p* = 0.0401. **f** a vs. b, *p* = 0.007, a vs. e, *p* ≤ 0.0001, a vs. f, *p* ≤ 0.0001. **g** Day 14, a vs. f, *p* ≤ 0.0001; Day 21, a vs. b, *p* ≤ 0.0001, a vs. e, *p* ≤ 0.0001, a vs. f, *p* ≤ 0.0001; Day 28, a vs. b, *p* ≤ 0.0001, a vs. e, *p* ≤ 0.0001, a vs. f, *p* ≤ 0.0001. **h** a vs. b, *p* ≤ 0.0001, a vs. e, *p* ≤ 0.0001, a vs. f, *p* ≤ 0.0001. *n* = 8 mice/group.

We first examined the pharmacokinetic effects of FA-L6 delivered as recombinant protein in vivo, and found that the half-life (~4 days) of FA-L6 (Fig. 5a) is similar to an IgG molecule^29^, indicating that FA-L6 is not rapidly cleared. Next, to investigate the safety and bone-building effects of FA-L6, we conducted a 28-day study in which 8-week-old female C57BL/6 mice (*n* = 8–10/treatment group) received twice weekly intraperitoneal injections with one of the following agents: vehicle (PBS, negative control), anti-green fluorescent protein (anti-GFP) antibody 10 mg/kg (negative control), anti-sclerostin antibody 10 mg/kg (positive control, subcutaneous), or FA-L6 (at increasing doses ranging from 0.001 to 1 mg/kg). No liver or gastrointestinal abnormalities outside normal range were observed for any treatment or dose level tested. In addition, in contrast to 18R5-DKK1c, no change in liver weight was observed following FA-L6 treatment. The liver weight increase with 18R5-DKK1c may be molecule specific or due to the AAV delivery method. Future studies will be needed to further understand this effect by 18R5-DKK1c. Treatment with FA-L6 resulted in dose-dependent increases in whole-body BMD, as measured by DEXA (Fig. 5b) and in proximal tibial BV/TV, as measured by micro-CT (Fig. 5c). As early as 7 days after treatment initiation with 1 mg/kg FA-L6, statistically significant elevations in BMD (Fig. 5b) and tibial BV/TV (Fig. 5c) were observed, and this effect was sustained through 21 days of longitudinal DEXA analysis. A significant increase in BMD was also observed after 0.3 mg/kg FA-L6 starting from day 14, and an increase in tibial BV/TV starting from day 7, with both effects sustained through day 21. Micro-CT of femurs on day 28 revealed that with FA-L6 treatment, BV/TV at the distal epiphysis of femurs was significantly increased (Fig. 5d), as was cortical thickness at the midshaft (Fig. 5e), suggesting improved resistance to fracture.

To understand the impact of narrowing the Fzd specificity to Fzd_1,2,7_ and effects of different Lrp binders on bone growth, we compared the effects of FA-L5, FA-L6, FB-L5, and FB-L6 at 1 or 10 mg/kg in naive 20-week-old female C57BL/6 mice (Supplementary Fig. 3) over a 14-day period. The control groups were vehicle (PBS), anti-GFP antibody control 10 mg/kg, and anti-sclerostin antibody 25 mg/kg. All treatments were administered twice weekly. All Wnt mimetics groups induced significant bone growth compared to vehicle, as shown by whole body as well as femur and lumbar BMD after 14 days of treatment (Supplementary Fig. 3) demonstrating that narrowing the Fzd specificity to Fzd_1,2,7_ remain effective in inducing bone growth. It is interesting to note that both FA and FB when attached with Lrp binder L5 showed trended better effects than fusions with L6 in the 10 mg/kg dose groups. Therefore, we focused on the Fzd binder with narrow Fzd specificity, FB (Fzd_1,2,7_ specific), fusions to L5, FB-L5 in subsequent studies.

The potential effects of Wnt mimetics on tissues outside of bone was assessed in naive 16-week-old female C57BL/6 mice with FA-L6 (Fzd_1,2,5,7,8_ specific) at 10 mg/kg and FB-L5 (Fzd_1,2,7_ specific) at 1 or 10 mg/kg over a 14-day period. The control groups were vehicle (PBS), anti-β-galactosidase (anti-β-gal, antibody control) 10 mg/kg, and anti-sclerostin antibody 25 mg/kg. All treatments were administered twice weekly. While the 10 mg/kg doses of FA-L6 and FB-L5 induced significant bone growth compared to vehicle, as shown by whole-body BMD after 14 days of treatment (Fig. 5f), liver and small intestine sections stained with proliferation marker, Ki67, revealed no differences across groups, and liver and intestine histology also appeared normal (Supplementary Fig. 4a, b).

We next investigated whether the positive effects on bone building can be applied to older animals. One-year-old female mice were treated twice weekly for 4 weeks with vehicle (PBS), anti-β-gal (antibody control) 10 mg/kg, anti-sclerostin antibody 25 mg/kg, or the Wnt mimetics FA-L6 or FB-L5 at two concentrations (1 or 10 mg/kg). FB-L5 10 mg/kg rapidly and significantly increased whole-body BMD compared to vehicle after only 14 days of treatment (Fig. 5g). FA-L6 10 mg/kg and anti-sclerostin antibody treatment also induced a significant increase in whole-body BMD compared to vehicle, however to a lesser degree and with delayed kinetics compared to FB-L5 (Fig. 5g). Micro-CT of femurs collected at day 28 revealed significant increases in distal femur epiphyseal BV/TV in the anti-sclerostin antibody, FA-L6 10 mg/kg, and FB-L5 10 mg/kg treatment groups, with the greatest overall gains observed in the FB-L5 cohort (Fig. 5h).

### Wnt mimetic induces gene expression in bone tissue

To further investigate the effects Wnt mimetics on Wnt signaling, cell proliferation, and osteogenesis, we examined gene expression changes in bone tissues. We administered a single intraperitoneal dose of either FB-L5 10 mg/kg or anti-β-gal (antibody control) to 12-week-old female C57BL/6 mice. Another control group of mice were injected with a single subcutaneous dose of anti-sclerostin antibody 25 mg/kg. Animals were euthanized 8, 24, 48, and 120 h after treatment, and marrow flushed tibias were isolated, and flash frozen for RNA extraction (Supplementary Table 3 and Supplementary Fig. 5). Compared to anti-β-gal, FB-L5 significantly increased the expression of genes produced predominantly by osteoblast lineages (*Runx2, Alp, Rankl*), similar to anti-sclerostin antibody. Osteoclast stimulating factor *Rankl* was induced transcriptionally in both the anti-sclerostin and FB-L5 groups. Compared to anti-sclerostin antibody, FB-L5 stimulated greater induction of *Axin2* and *Mki67*, suggesting that bone accrual mediated by FB-L5 treatment occurs differently than that mediated by anti-sclerostin antibody treatment, possibly due to the direct activation of Wnt signaling and downstream cascade.

### Wnt mimetic corrects low bone mass in an osteoporosis model

**Fig. 6:**
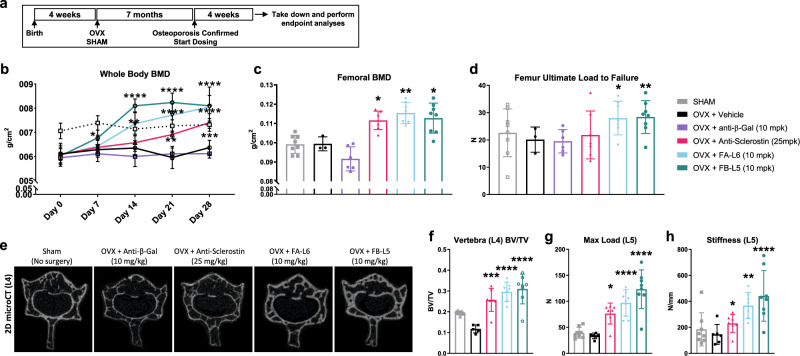
Wnt mimetic antibodies reverse bone loss associated with ovariectomy induced osteoporosis. **a** Schematic of experimental design. 4-week-old C57BL6/J female mice were surgically ovariectomized (OVX). Seven months later, DEXA was used to confirm bone loss in the OVX groups prior to treatment initiation. Mice were injected bi-weekly with intraperitoneal administration of FA-L6 10 mg/kg or FB-L5 10 mg/kg antibody, or a negative (anti-β-gal) or positive control (bi-weekly subcutaneous injection of anti-sclerostin antibody 25 mg/kg) for 28 days. **b** Longitudinal DEXA measurements were collected and whole-body BMD quantified for days 0, 7, 14, 21, and 28 and **c** whole femur BMD on day 28. Bone strength at the mid-shaft femur was analyzed by a standard 3-point bending test and quantified for **d** average ultimate load to failure. **e** Representative micro-CT reconstructions of day 28 L4 lumbar vertebra (images from *n* = 8 samples/group having similar results) and **f** quantification of L4 BV/TV. Compressive loading was applied to L5 vertebrae to quantify **g** max load and **h** stiffness. Graphs represent mean values ± SD, *n* = 8 mice/group, except Vehicle + OVX, where *n* = 3. Statistical significance was determined by two-way ANOVA for **b** and one-way ANOVA for all others **b**–**d** and **f**–**h**, where the following letters define groups (a = OVX + Vehicle, b = OVX + Anti-Sclerostin, c = OVX + FA-L6, d = OVX + FB-L5) and *p < 0.05, **p < 0.01, ***p < 0.001, ****p < 0.0001. **b** Day 7, a vs. d, *p* = 0.0135. Day 14, a vs. c, *p* = 0.0014, a vs. d, *p* ≤ 0.0001. Day 21, a vs. b, *p* = 0.0023, a vs. c, *p* ≤ 0.0001, a vs. d, *p* ≤ 0.0001. Day 28, a vs. b, *p* = 0.0005, a vs. c, *p* ≤ 0.0001, a vs. d, *p* ≤ 0.0001. **c**, a vs. b, *p* = 0.0427, a vs. c, *p* = 0.0023, a vs. d, *p* = 0.0156. **d**, a vs. c, *p* = 0.0164, a vs. d, *p* = 0.0098. **f** a vs. b, *p* = 0.0002, a vs. c, *p* ≤ 0.0001, a vs. d, *p* ≤ 0.0001. **g** a vs. b, *p* = 0.0111, a vs. c, *p* ≤ 0.0001, a vs. d, *p* ≤ 0.0001. **h** a vs. b, *p* = 0.0174, a vs. c, *p* = 0.0056, a vs. d, *p* ≤ 0.0001.

To extend our understanding of how Wnt mimetic treatment may be relevant in a skeletal disease setting, we utilized C57BL/6 female mice that had undergone ovariectomy or sham surgery at 4 weeks of age (Fig. 6a). At 8 months of age, DEXA was performed to confirm that hormone ablation had led to an osteoporotic phenotype (Fig. 6b, Day 0, sham vs. all other groups). Once low bone mass had been established, we randomized groups based on equivalently decreased BMD and initiated twice weekly treatments for 4 weeks with vehicle (PBS), anti-β-gal 10 mg/kg (antibody control), anti-sclerostin antibody 25 mg/kg, or the Wnt mimetics FA-L6 10 mg/kg or FB-L5 10 mg/kg. As shown by whole-body BMD (measured by DEXA), two treatments of FB-L5 in the first week were able to significantly rescue the osteoporotic phenotype caused by ovariectomy (Fig. 6b, Day 7). Reversal of bone loss was also observed with FA-L6 (beginning by day 14) and anti-sclerostin antibody (beginning by day 21) (Fig. 6b). However, the Wnt mimetics, particularly FB-L5, reversed bone loss and enhanced bone accrual in ovariectomy mice to a level higher than that observed in sham control animals. This heightened BMD rose rapidly by day 14 and was sustained through day 28, similar to what was observed in both young and old animal models (Fig. 5f–h).

At day 28, femurs and vertebrae were collected for microarchitectural analysis and biomechanical strength and treatments compared to OVX + vehicle control. FB-L5 treatment significantly increased femoral BMD (Fig. 6c). Also, biomechanical testing of femurs revealed that both FA-L6 and FB-L5 treatment significantly increased ultimate load to failure compared to OVX + vehicle control (Fig. 6d). Vertebrae (L4) from micro-CT reconstructions (Fig. 6e) demonstrated visible increases in both cortical and cancellous bone growth, which were confirmed by the statistically significant increases in BV/TV following treatment with FA-L6, FB-L5, or anti-sclerostin antibody (Fig. 6f). Each of these treatments also showed statistically significant increases in maximum load (Fig. 6g) and stiffness (Fig. 6h), as shown by compressive force biomechanical testing of lumbar vertebrae (L5). This striking increase in trabecular and cortical bone of both the femur and vertebrae in ovariectomized mice suggests that Wnt mimetics, particularly FB-L5, can rapidly reverse severe bone loss due to hormone ablation-induced osteoporosis.

### Combination or sequential therapy with Wnt mimetic improves bone mass

**Fig. 7:**
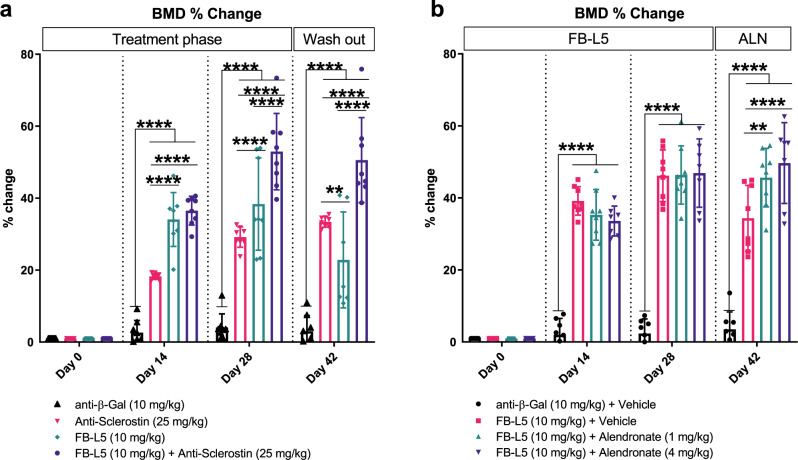
Combination studies of Wnt mimetic antibodies with currently available therapeutics. **a** 12-week-old C57BL6/J female mice were injected bi-weekly for 28 days with intraperitoneal administration of FB-L5 (10 mg/kg) antibody and compared to a negative control anti-β-gal (10 mg/kg) and positive control bi-weekly subcutaneous administration of 25 mg/kg anti-sclerostin antibody or a combination of anti-sclerostin antibody and FB-L5. A 2-week washout period then followed (day 42 assessment). Longitudinal DEXA measurements were collected and whole-body % BMD change quantified for days 0, 14, 28, and 42, normalized to baseline. At day 28, combination treatment induced statistically significant increases in BMD compared to individual treatments. At day 42, combination treatment maintained the bone accrual gained during the first 4 weeks of treatment and remained statistically significant compared to individual treatments. **b** 12-week-old C57BL6/J female mice were injected bi-weekly for 28 days with intraperitoneal administration of FB-L5 (10 mg/kg) antibody and compared to a negative control anti-β-gal (10 mg/kg). From day 28 through day 42, animals were treated with either vehicle or alendronate at 1 or 4 mg/kg. Longitudinal DEXA measurements were collected and whole-body % BMD quantified for days 0, 14, 28, and 42, normalized to baseline. **a**, **b** Graphs represent mean values ± SD, *n* = 8 mice/group. Statistical significance determined by two-way ANOVA (**p < 0.01, ****p < 0.0001), where the following letters define groups, **a** (a = anti-β-Gal, b = Anti-Sclerostin, c = FB-L5, d = FB-L5 + Anti-Sclerostin) and **b** (a = anti-β-Gal + Vehicle, b = FB-L5 + Vehicle, c = FB-L5 + Alendronate (1 mg/kg), d = FB-L5 + Alendronate (4 mg/kg)). **a** Day 14, a vs. b, *p* ≤ 0.0001, a vs. c, *p* ≤ 0.0001, a vs. d, *p* ≤ 0.0001, b vs. c, *p* ≤ 0.0001, b vs. d, *p* ≤ 0.0001. Day 28, a vs. b, *p* ≤ 0.0001, a vs. c, *p* ≤ 0.0001, a vs. d, *p* ≤ 0.0001, b vs. c, *p* ≤ 0.0001, b vs. d, *p* ≤ 0.0001, c vs. d, *p* ≤ 0.0001. Day 42, a vs. b, *p* ≤ 0.0001, a vs. c, *p* ≤ 0.0001, a vs. d, *p* ≤ 0.0001, b vs. c, *p* = 0.0031, b vs. d, *p* ≤ 0.0001, c vs. d, *p* ≤ 0.0001. **b** Day 14, a vs. b, *p* ≤ 0.0001, a vs. c, *p* ≤ 0.0001, a vs. d, *p* ≤ 0.0001. Day 28, a vs. b, *p* ≤ 0.0001, a vs. c, *p* ≤ 0.0001, a vs. d, *p* ≤ 0.0001. Day 42, a vs. b, *p* ≤ 0.0001, a vs. c, *p* ≤ 0.0001, a vs. d, *p* ≤ 0.0001, b vs. c, *p* = 0.0023, b vs. d, *p* ≤ 0.0001.

We investigated whether Wnt mimetic and anti-sclerostin antibody treatment might work in a combinatorial way. We treated 12-week-old naive animals with anti-sclerostin antibody 25 mg/kg and FB-L5 10 mg/kg, either alone or in combination, twice weekly for 4 weeks, followed by a 2-week washout period of no treatment. In the first 4 weeks, combination treatment induced statistically significant increases in BMD versus individual treatments (Fig. 7a). Furthermore, after the 2-week washout period, combination therapy maintained the bone accrual gained during the first 4 weeks of treatment and remained statistically significant compared to individual treatments. Taken together, these results suggest that a combination of anti-sclerostin antibody and Wnt mimetic treatment work complementarily to elicit prolonged bone accrual and/or reduced bone resorption.

We next examined whether sequential treatment of antiresorptive bisphosphonate can help preserve bone mass gained by FB-L5 treatment. We treated 12-week-old naive animals with twice weekly intraperitoneal injections of FB-L5 10 mg/kg alone for 4 weeks and then either discontinued treatment or administered alendronate at two different doses, 1 or 4 mg/kg (given as a single oral gavage dose) (Fig. 7b). With the addition of either dose of alendronate, bone accrual induced by FB-L5 at day 28 was maintained at day 42. BMD at day 42 was significantly increased in the group that received combined sequential treatment compared to FB-L5 alone.

### Wnt mimetics as delayed treatment for fracture repair

**Fig. 8:**
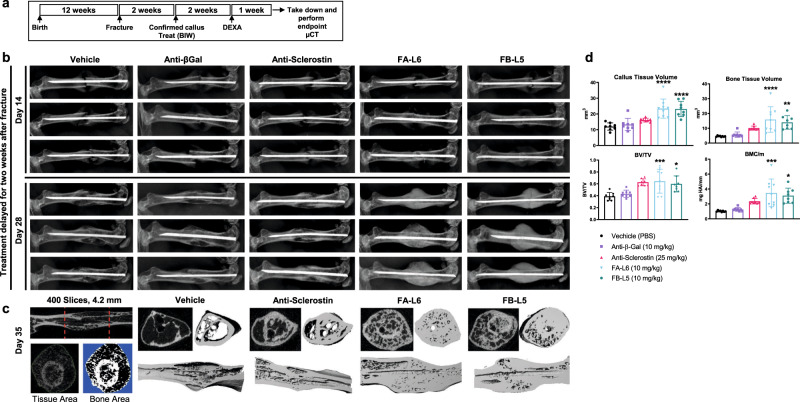
Wnt mimetic antibodies increase bone accrual and bone repair when provided 2 weeks after fracture. **a** Schematic of experimental design. 12-week-old C57BL6/J female mice were surgically fractured adhering to the Einhorn fracture model. Animals did not receive any treatments for 2 weeks to allow for a normal cartilaginous callus to form. **b** Callus formation was visually confirmed by x-ray (top panel) at day 14; representative images (*n* = 8 samples/group having similar results) demonstrate a small, but visible, callus with no cortical fusion. Mice were injected bi-weekly (BIW) for 21 days with intraperitoneal administration of FB-L5 (10 mg/kg), FA-L6 (10 mg/kg) and compared to a negative control anti-β-gal and bi-weekly subcutaneous administration of anti-sclerostin (30 mg/kg) antibody. Representative x-ray images demonstrate the large amount of cortical fusion and radiographic contrast present in the callus and marrow cavity of bones treated with FA-L6 and FB-L5 (bottom panel) at day 28. **c** Femurs were isolated at day 35 and scanned by µCT. Measurements were made according to the left panel, where red dotted lines represent 4.2 mm, or 400 slices by µCT, centered on the fracture line with image analysis shown for tissue area vs. bone area, and representative images shown for each treatment group. **d** Callus tissue volume, bone tissue volume, BV/TV, and bone mineral content/mm (BMC/mm) were measured for all animals at day 35. **d** Graphs represent mean values ± SD, *n* = 8 mice/group. Statistical significance was determined by one-way ANOVA (*p < 0.05, **p < 0.01, ***p < 0.001, ****p < 0.0001), where the following letters define groups, (a = Vehicle, b = FA-L6, c = FB-L5). Callus tissue volume, a vs. b, *p* ≤ 0.0001, a vs. c, *p* ≤ 0.0001. Bone tissue volume, a vs. b, *p* = 0.0001, a vs. c, *p* = 0.0022. BV/TV, a vs. b, *p* = 0.001, a vs. c, *p* = 0.0116. BMC/mm, a vs. b, *p* = 0.001, a vs. c, *p* = 0.0117.

Wnt signaling has been shown to regulate chondrocyte and cartilage differentiation^30^, which is necessary for callus development in early fracture repair. Considering the dramatic effects of Wnt mimetic treatment in our previous experiments, we wanted to determine if fracture repair could be enhanced. We first examined the effect of adding a Wnt mimetic immediately after fracture, and found that callus formation and subsequent fracture repair was inhibited (Supplementary Fig. 6). Based on these results, we reasoned that it would be better to activate Wnt signaling after the initial phase of fibrocartilaginous callus formation. Thus, we examined the role of delayed treatment with Wnt mimetics in fracture healing, utilizing the Einhorn mouse model of a femoral fracture^31^. The experimental design is shown in Fig. 8a. Two weeks after fracture, callus formation was confirmed by x-ray for all animals (Fig. 8b, top panel), and mice were randomized into five groups for treatment with vehicle (PBS), anti-β-gal antibody 10 mg/kg, anti-sclerostin antibody 25 mg/kg, FA-L6 10 mg/kg, or FB-L5 10 mg/kg twice weekly for 3 weeks. Two weeks after initiating protein treatment (4 weeks after fracture), radiographic contrast of the callus was increased in animals treated with either FA-L6 or FB-L5 (Fig. 8b, bottom panel). After 1 additional week of treatment, we excised bones and performed micro-CT to quantify tissue and bone volume (Fig. 8c). Statistically significant increases were observed in callus tissue volume, bone tissue volume, BV/TV, and BMC/mm in femurs from animals treated with FA-L6 and FB-L5 compared to vehicle (Fig. 8d). These results demonstrate that Wnt mimetics can stimulate rapid and robust mineralization in healing bone tissue after fracture.

## Discussion

The main objective of the series of studies reported here was to test the hypothesis that Fzd-Lrp receptor heterodimerizing Wnt mimetics have the ability to activate Wnt/β-catenin signaling to stimulate rapid bone growth in vivo. Building on previous in vitro findings^26^, we used a mouse model to first investigate whether a Wnt mimetic agonist could stimulate bone accrual in vivo. We demonstrated that systemic delivery of a Wnt mimetic (namely, 18R5-DKK1c) enhances bone-building activities and that the resulting bones were functional and biomechanically sound. Having demonstrated this proof-of-concept, we then developed Wnt mimetics in a bi-specific IgG antibody format with biophysical properties suitable for therapeutic delivery and tested the bone-building effects of these agonists in normal and osteoporotic murine models. In our studies, the Wnt mimetics produced rapid bone growth effects. Whole-body BMD and proximal tibial BV/TV were significantly increased, generally within 7–14 days following treatment, with effects sustained through the 28-day study periods. These positive effects were observed in younger (8 and 16 weeks old) and older (1-year-old) female mice. Likewise, in hormone ablation-induced osteoporosis, the Wnt mimetics increased whole-body and femoral BMD as well as vertebral cortical and cancellous bone growth. Furthermore, biomechanical testing revealed significant increases in bone strength. These Wnt molecules not only built bone rapidly in the animal models, but also showed a sustained effect on bone accrual when combined with currently available antiresorptive and bone anabolic medications. While anti-sclerostin treatment was also shown to reverse bone loss, the effect was delayed compared to Wnt mimetics at the doses tested. Future broad dose range studies comparing the bone accrual effects of anti-sclerostin and Wnt mimetics may provide further insights on the similarities and differences between these treatments.

We and others have shown that numerous Fzds are expressed in bone tissues or isolated cell lines^32,33^. It remains to be determined, however, which specific Fzd(s) have the more profound functional impact on bone accrual. Our results from 18R5-DKK1c, FA-L5, FA-L6, FB-L5, and FB-L6 suggest that multiple Fzds and Fzd subfamilies can confer bone-building effects. To further understand Fzd and Lrp specificities, additional agonists with various binders of Fzd_1,2,7_ or Fzd_5,8_, or mono-specific Fzd, coupled with various binders to Lrp5 or Lrp6, need to be engineered and examined for their differential effects on bone-building and fracture repair. Similarly, the resulting bi-specific agonists with different activities in the Wnt signaling reporter assay and in vivo bone efficacy need to be thoroughly investigated to elucidate functional impacts of Fzd specificities. It is possible that other Fzd-Lrp combinations will yield even greater bone accrual. Furthermore, skeletal sites may differ in their sensitivity to particular Fzd-Lrp combinations, a possibility that would require additional studies. In addition, under certain skeletal disease conditions, there could be either an abundance or lack of a particular Fzd subfamily. These exciting possibilities could provide a therapeutic armamentarium for targeting a variety of skeletal diseases or fractures at different sites.

Current treatments for osteoporosis include antiresorptive therapies (bisphosphonates, estrogens, selective estrogen receptor modulators, calcitonin, and monoclonal antibodies such as denosumab) and anabolic therapies (teriparatide, abaloparatide, and the anti-sclerostin monoclonal antibody romosozumab, which indirectly activates Wnt signaling by blocking a negative regulator, sclerostin). Sclerostin acts as a direct antagonist to Wnt signaling through binding Lrp5 and Lrp6^34^. Combination or sequential therapy has the potential to improve bone mass and bone strength compared to monotherapy^9,35,36^. In our studies, we found that the combination of our Wnt agonist FB-L5, with bisphosphonate or anti-sclerostin antibody treatment induced greater improvements in BMD compared to monotherapy. These results suggest that Wnt mimetics in combination with current clinical treatments for osteoporosis may enhance bone repair and help maintain bone mass. Wnt mimetics may also provide a stimulus for rapidly improving bone mass in pre-optimization of patients preparing for skeletal surgery.

The mechanism of how Wnt signaling activation propels bone formation remains to be elucidated. Bone formation and homeostasis is a balance between osteoblast and osteoclast activities. Studies suggest that Wnt activation possibly impacts osteoblasts and osteoblast lineages by increasing the number of osteoblasts and bone-lining cells, and by enhancing activity and differentiation of osteoblasts^37^. In our study, we observed increased osteoblast number in vivo, consistent with what has been previously reported in the literature in vitro^26^. Using gene expression analysis, we found that Ki67 signals were the most profoundly elevated gene signature after single-dose FB-L5 treatment, providing insight into the rapid bone growth that we detected radiographically. Likewise, we observed a rapid return to baseline in the Ki67 expression level. It is not clear whether longer-term treatment with a Wnt mimetic would result in the same magnitude of amplification in Ki67, or whether there exists an internal rheostat that would diminish continued proliferation. Of course, while the increase in Ki67 does strongly suggest enhanced proliferation, additional experiments and analyses are required to unequivocally prove this. Perhaps a renewed pool of osteoblast precursors would be required before additional stimulation of proliferation could be achieved. However, further studies are needed to delineate the precise mechanism of action. The effect of Wnt signaling on osteoclasts is less clear. Some research suggests a direct effect on osteoclasts^38^, but most studies point to an indirect effect through Wnt signal regulation of OPG and RANKL expression^19^. We did not observe either a profound impact of the Wnt mimetic agonist 18R5-DKK1c on osteoclasts based on TRAP staining or a sustained increase in the expression level of RANKL. From our study of the Wnt mimetic FB-L5 in combination with bisphosphonate, it is clear that some of the bone accrual induced by Wnt mimetic treatment is lost if there is no bisphosphonate present. One possible explanation is that animals dosed over a few weeks develop neutralizing anti-drug antibodies, similar to what has been previously reported^39^, effectively diminishing the initial stimulatory activity of the Wnt mimetics on bone accrual. However, we did not comprehensively study the development of anti-drug antibodies or osteoclasts and the key factors affecting osteoclast differentiation and activation. Future studies will need to examine the in vitro and in vivo direct effects of Wnt mimetic treatment on the fusion, maturation, and activity of osteoclasts.

Bone regeneration and fracture repair are highly complex, involving MSCs and multiple signaling pathways, including the Wnt/β-catenin signaling pathway^40^. During fracture healing, β-catenin and Wnt ligands and receptors, including Fzd_1,2,4,5_ and Lrp 5 and 6, are upregulated^41,42^. Wnt/β-catenin signaling has been shown to control osteoblast and chondrocyte differentiation^30^. We found that administration of a Wnt mimetic immediately after fracture inhibited callus formation and subsequent repair. These resutls were surprising beause of the accompanying increase in mineralization of the fractured bone extending away from, but clearly absent in, the fracture site. It appears that exogenous Wnt stimulation at the time of frature actively inhibits the formation of cartilagenous callus while also mobilizing osteoblast precursors within the marrow cavity to rapidly mineralize, however this mechanism remains to be clarified. Wnt mimetic treatment initiated 2 weeks post-fracture for a total of 3 weeks, resulted in significant increases in callus and bone tissue volumes as well as BMD, suggesting a possible therapeutic role for Wnt mimetics in healing bone tissue after fracture^43^. Follow-up studies will need to focus on how the highly mineralized, repaired callus will remodel and respond to washout under normal loading conditions. Additionally, the increases in contralateral, unfractured femoral BMD could provide rapid protection against subsequent fracture. It will be interesting to expand upon these findings to understand whether local injection of a Wnt mimetic is efficacious in building bone after fracture or following skeletal reconstructive therapy.

In conclusion, our studies of Wnt mimetic molecules provide a blueprint to design a fast-acting bone anabolic therapeutic candidate that can benefit individuals with severe bone deficiency as well as those who need to build bone quickly. The robust efficacy and short-term treatment requirement for our molecule may be beneficial as well from a safety perspective. Combination of a Wnt mimetic with antiresorptive treatment to maintain bone gain may be another attractive treatment scenario. Our emerging toolbox of Wnt mimetics with different properties and receptor specificities will allow us to engineer a candidate molecule that has increased selectivity for bone, while deepening our understanding of the biology of Wnt activation and bone mass regulation. Wnt signaling mutations can lead to many diseases, especially activating mutations in intracellular signaling components which have been linked to cancer. Anti-sclerostin approach has the advantage of more limiting off-target effects due to the relatively specific bone expression of sclerostin while the Wnt mimetics described here are applied systemically. Even though there are some differences between Wnt mimetics and mutations found in cancer, for example, Wnt mimetics induced signaling may differ from intracellular activating mutations in amplitude and duration, as Wnt mimetics activate only a subset of Fzds and Wnt mimetics do not directly affect R-Spondins to further enhance signaling. In addition, negative feedback regulatory components are still active which could limit mimetics activities and applying bone targeting approaches to Wnt mimetics may reduce systemic effects of such molecules. Nonetheless, thorough safety studies for Wnt mimetics will need to be conducted to demonstrate appropriate benefit versus risk profile for future therapeutic considerations.

## Methods

### Study design

The objectives of the studies reported here were: (1) to determine whether 18R5-DKK1c can be delivered systemically to induce bone accrual in vivo, (2) to engineer bi-specific molecules with different Fzd and Lrp binders and specificities and determine the efficacy of these Wnt mimetics on building bone in young and older uninjured mice and in an ovariectomized mouse model of osteoporosis, (3) determine whether Wnt mimetics induce additive bone effects when combined with currently available antiresorptive or anabolic therapies, and (4) determine the ability for Wnt mimetics to improve fracture healing in a mouse long bone fracture model. Wild-type female C57BL/6 mice (The Jackson Laboratory) were utilized (*n* = 8–10/group) in all experiments. All mice were naive animals, except those that underwent ovariectomy or sham surgery. No power analyses were used to calculate sample sizes, and no samples were excluded. All experiments were conducted under non-blinded conditions. Pathology assessment was performed under blinded conditions.

### Animal studies and study treatments

All animal experiments were performed according to national ethical guidelines in addition to the guidance and approval by the Institutional Animal Care and Use Committee (IACUC) of Surrozen, Inc. Euthanasia was conducted in compliance with the current requirements of The Guide for the Care and Use of Laboratory Animals, 8^th^ Edition, and the American Veterinary Medical Association (AVMA) Guidelines on Euthanasia. All animals were obtained from the Jackson Laboratory (Bar Harbor, ME). On arrival, animals were randomly assigned to group (5 animals/cage) housing and provided with rodent diet and water ad libitum. All mice were maintained on a 12:12-h light/dark photoperiod at an ambient temperature of 22 ± 2 °C. All antibodies used in the experiments were manufactured at Surrozen, Inc. laboratory and delivered via intraperitoneal injection. The anti-sclerostin antibody used in our experiments was prepared based on published sequence data^44^ and administered via subcutaneous administration. Alendronate sodium (Fosamax^®^, Merck & Co., Inc., Whitehouse Station, NJ) was purchased from a local pharmacy, dissolved in PBS, and delivered as oral gavage.

### Einhorn fracture model

For fracture studies, an adapted fracture method first described by Bonnarens and Einhorn^31^ was performed. Animals were maintained under anesthesia with ketamine and xylazine throughout the procedure and all animals were given a dose of buprenorphine prior to surgery. After making an anterior longitudinal midline incision centered over the knee joint, a 30-gauge needle was used as an intramedullary pin, driving it through the distal femur toward the femoral head after subluxation of the patella. The needle was clipped and gently nested into the distal femur to avoid soft tissue damage. Pin insertion was confirmed radiographically prior to fracture. Fracture is generated by dropping a weight onto the femur using a custom guillotine style fracture device. The weight was determined as the amount of weight needed from a defined height to generate an impact leading to a focal transverse fracture at the mid-diaphysis.

### Micro-computed tomography

Micro-computed tomography of murine trabecular and cortical bone was completed using a Scanco vivaCT 40 scanner (Scanco Medical, Brüttisellen, Switzerland). Distal femurs, proximal tibiae, and lumbar samples were scanned with a 10 µM isotropic voxel size according to recommended guidelines^45^. Sample volumetric analysis was performed using Scanco software. For the distal femur and proximal tibia, 100 transverse CT slices were evaluated proximal to the growth plate. For vertebrae, trabecular bone was evaluated between the cranial and caudal growth plate.

### Dual energy X-ray absorptiometry (DEXA)

BMD of animals was measured via an in vivo DEXA method using a Faxitron UltraFocus (Faxitron Bioptics, Tucson, Arizona). Animals were anesthetized during imaging through isoflurane, in accordance with an approved Surrozen, Inc. IACUC protocol. Sample regions of interest (ROI) included the entire murine skeleton, except material above the cervical spine due to increased radiographical intensity of the skull. BMD and bone mineral content (BMC) were calculated using the accompanying Faxitron software.

### Gene expression analysis

At time zero, female C57BL/6 mice were injected with one of the following proteins: negative control antibody, anti-sclerostin antibody, or Wnt mimetic. At described times (Supplementary Fig. 5), animals were euthanized, and tibias collected. Soft tissue and proximal and distal epiphyses were removed prior to flushing of the medullary cavity with PBS to remove bone marrow. The remaining shaft was then flash frozen in liquid nitrogen until further processing. Using the Qiagen TissueLyser and Qiagen 5 mm Stainless Steel Beads, samples were homogenized in 1 mL Qiagen Trizol lysis reagent until complete homogenization. RNA was isolated via acid guanidinium thiocyanate-phenol-chloroform technique and purified with Qiagen RNeasy Plus Mini spin columns, including DNase digestion per manufacturer’s instructions. A 1 µg sample of RNA was reversed transcribed to cDNA using Invitrogen SuperScript IV VILO Master Mix, in accordance with the manufacturer’s protocol. Quantitative-PCR was completed with Applied Biosystems Taqman probes and Applied Biosystems Taqman Fast Advanced Master Mix for 40 cycles. Analysis was performed using the 2^−ddCt^ method. All primers used can be found in the Supplementary Table 4.

The following method was used for human and mouse femur bone (Fig. 1d, e). Fresh human cadaveric femur tissue was purchased from vendor (Folio Conversant). Femur heads were shipped on wet ice within 24 h of death and tissue used immediately upon receipt. In a tissue culture hood, kimwipes and scissors were used to remove connective tissue before removing the femoral head. The remaining diaphysis was cut into 1–2 mm small pieces with a pair of dissecting scissors. Bone pieces were washed with cold PBS by inverting and shaking before being allowed to settle, then the supernatant was removed, and the process repeated 5 times or until the bone pieces were white with no remaining connective tissue or vasculature remaining. The process outlined above for tibias was used from this point forward. Values were calculated using the delta CT method and values are represented as expression relative to housekeeping gene actin multiplied by a factor of 1000.

### Super TopFlash (STF) assay

Wnt signaling activity was measured using HEK293 cells containing a luciferase gene controlled by a Wnt-responsive promoter (Super TopFlash reporter assay), as previously reported^26^. In brief, cells were seeded at a density of 10,000 per well in 96-well plates 24 h prior to treatment at the presence of 3 μM IWP2 to inhibit the production of endogenous Wnt ligands. Recombinant WNT3A (R&D systems) was used as a positive control. Cells were lysed with Luciferase Cell Culture Lysis Reagent (Promega), and activity was measured with the Luciferase Assay System (Promega), using vendor suggested procedures. Data were plotted as average −/+ standard deviation of triplicates and fitted by non-linear regression using Prism (GraphPad Software, San Diego, CA).

### Affinity measurements

Binding kinetics of FA IgG, FB IgG, L5 (FB-L5), and L6 (FB-L6) to each CRD of Fzd_1,2,5,7,8_, Lrp5 (CREATIVE BIOMART, Shirley, NY) or Lrp6E3E4^27^ were determined by bio-layer interferometry (BLI) using Octet Red 96 (PALL ForteBio, Fremont, CA) instrument at 30 °C, 1000 rpm with anti-human IgG Fc capture (AHC) biosensors. FA IgG, FB IgG, FB-L5, or FB-L6 diluted to 50 nM in the running buffer (PBS, 0.05% Tween-20, 0.5% BSA, pH 7.2) were captured to the AHC biosensor followed by dipping into wells containing the relevant Fzd CRDs or Lrp proteins at different concentrations in running buffer or into a well with only running buffer as a reference channel. K_D_ for each binder was calculated by Octet System software, based on fitting to a 1:1 binding model. Binding specificities of FA IgG and FB IgG to 10 Fzds were also examined by the BLI assay. Biotinylated Fzd CRDs^27^ diluted to 50 nM in the running buffer were captured to the SA biosensor followed by dipping into wells containing the FA IgG or FB IgG at 200 nM in running buffer.

### Serum bone formation marker analysis

Blood samples were obtained at days 7, 14, 21, and 28 of the treatment phase for both treated and control animals. All blood was collected 24 h after the most recent test article treatment. Serum was obtained from blood samples through centrifugation in MiniCollect^®^ Tube 0.8 ml Z Serum Separator tubes (Greiner Bio-One). Serum samples were then assessed for amount of procollagen type-1 N-terminal propeptide (P1NP) using the Rat/mouse P1NP EIA kit (Immunodiagnostic Systems, Boldon, UK), per manufacturer protocol. The bone-anabolic effect was then assessed by comparing the P1NP levels of treated versus negative-control treated animals.

### Anti-hFc binding enzyme-linked immunosorbent assay

Plates were coated with anti-human IgG at 1 μg/mL in PBS (AffiniPure Goat Anti-Human IgG, Fcγ fragment specific) Jackson ImmunoResearch, West Grove, PA, USA) and incubated at 4 °C overnight. Plates were then washed with 1x PBS with 0.05% Tween-20 (PBST) and blocked using SuperBlock T20 (PBS) Blocking Buffer (ThermoFisher Scientific). Each plate was then incubated at 37 °C for 1 h and washed with PBST. Bi-specific antibody was used to create a standard curve, and standards and samples diluted in PBST were incubated at room temperature for 1 h. Plates were washed with PBST, and Peroxidase AffiniPure Donkey Anti-Human IgG, Fcγ fragment specific (Jackson ImmunoResearch) was applied at room temperature for 1 h. Plates were washed with PBST, and 1-Step Ultra TMB-ELISA Substrate Solution (ThermoFisher Scientific) was applied for 4 min. The TMB reaction was stopped with sulfuric acid solution and the plate was measured for absorbance at 450 nm in a SpectraMax^®^ Paradigm^®^ (Molecular Devices). A standard curve was created, and serum concentrations were determined in the accompanying software.

### Histology of murine specimens

Tissues were extracted and immediately fixed in 10% neutral buffered formalin for 24 h prior to extensive wash in water. Skeletal samples were then incubated in 10% di- and tetra-sodium EDTA until complete decalcification, followed by serial ethanol dehydration and paraffin embedding. Separate tissues taken for histopathology were placed in 70% ethanol until paraffin embedding following formalin fixation. After embedding, the tissues underwent routine hematoxylin and eosin staining for analysis. Fluorochrome labeling of the bones was performed by intraperitoneal injections of calcein (30 mg/kg) (Sigma Chemical, St. Louis, MO, USA) administered 8 days prior to sacrifice. Formalin fixed tibias were embedded in methyl methacrylate and Goldner’s trichrome and Tartrate Resistant Acid Phosphatase staining was performed on skeletal samples for histomorphometry analysis, and blinded quantification of the endosteal proximal tibia was performed using OsteoMeasure (OsteoMetrics, Atlanta, GA, USA).

### Immunofluorescence staining

For the characterization of Ki-67 expressing cells in liver and intestine, formalin fixed paraffin embedded tissue sections were examined. All samples were fixed in 10% buffered formalin. The primary Ki67 rat anti-mouse antibody (ThermoFisher) was diluted to 1:300 with PBS and incubated with the slides overnight at 4 °C. After washing, Ki67-labeled donkey anti-rat IgG (ThermoFisher), diluted 1:500 with PBS, served as secondary antibody. Slides were examined with a Leica (Wetzlar, Germany) DMi8 inverted microscope. Digital images were obtained with a digital-camera system (sCMOS camera DFC9000, Leica).

### Mechanical testing

Femur and vertebra bone strength was analyzed by a modified, published method^46^. Three-point bending was used to evaluate the biomechanical strength of femur. The femur was thawed from frozen stage (−20 °C) to room temperature. The attached muscles and tendons were removed. The femur was wetted with saline soaked gauze. Using MTS 858 Mini Bionix II (MTS, Eden Prairie, MN, USA), the femur was set, and force-advanced at 6 mm/min until immediately after the load peak. Vertebrae were assessed by standard compressive force analysis. The L4 was thawed to room temperature and prepared as described in the 3-point test. The force was advanced at 20 mm/min until immediately after the load peak. The associated TestWork^TM^ (Version 4.08) automatically recorded the advancement of force and resulting displacement recorded by blinded technician.

### Statistics

Data were analyzed using Prism (GraphPad Software, San Diego, CA, USA) via a one-way or two-way ANOVA (where appropriate) to determine significant differences between treatment versus basal, wild-type, or negative-control groups. Post-hoc analyses were performed using Tukey’s method in the accompanying software. *P* values <0.05 were considered statistically significant, and are indicated in the figures with an asterisk (*), unless otherwise reported.

### Reporting summary

Further information on research design is available in the Nature Research Reporting Summary linked to this article.

## Supplementary information

Supplementary Information

Reporting Summary

## Data Availability

The authors declare that the data supporting the findings of this study are available within the paper and its Supplementary Figures. Source data are provided with this paper.

## Source data

Source Data

## Material availability

All unique/stable reagents generated in this study are available from the corresponding authors with a completed Materials Transfer Agreement.

## References

[1] Pedersen AB, Ehrenstein V, Szepligeti SK, Sorensen HT (2017). Excess risk of venous thromboembolism in hip fracture patients and the prognostic impact of comorbidity. Osteoporos. Int.

[2] Toth, E. et al. History of Previous Fracture and Imminent Fracture Risk in Swedish Women Aged 55 to 90 Years Presenting With a Fragility Fracture. *J. Bone Miner. Res*. 10.1002/jbmr.3953 (2020). 10.1002/jbmr.3953 PMC9328134 31914206

[3] Borhan S (2019). Incident Fragility Fractures Have a Long-Term Negative Impact on Health-Related Quality of Life of Older People: The Canadian Multicentre Osteoporosis Study. J. Bone Min. Res.

[4] Silva DMW (2019). Incidence and excess mortality of hip fractures in a predominantly Caucasian population in the South of Brazil. Arch. Osteoporos..

[5] Bliuc D, Center JR (2016). Determinants of mortality risk following osteoporotic fractures. Curr. Opin. Rheumatol..

[6] Lippuner K, Johansson H, Kanis JA, Rizzoli R (2009). Remaining lifetime and absolute 10-year probabilities of osteoporotic fracture in Swiss men and women. Osteoporos. Int..

[7] Oden A, McCloskey EV, Kanis JA, Harvey NC, Johansson H (2015). Burden of high fracture probability worldwide: secular increases 2010-2040. Osteoporos. Int..

[8] Compston JE, McClung MR, Leslie WD (2019). Osteoporosis. Lancet.

[9] Langdahl, B. L. Overview of treatment approaches to osteoporosis. *Br J Pharmacol*. 10.1111/bph.15024 (2020). 10.1111/bph.15024 32060897

[10] Khosla S, Shane E (2016). A crisis in the treatment of osteoporosis. J. Bone Min. Res..

[11] Marini JC (2017). Osteogenesis imperfecta. Nat. Rev. Dis. Prim..

[12] Bloomfield SA (2010). Disuse osteopenia. Curr. Osteoporos. Rep..

[13] van Amerongen R, Nusse R (2009). Towards an integrated view of Wnt signaling in development. Development.

[14] Niehrs C, Acebron SP (2012). Mitotic and mitogenic Wnt signalling. EMBO J..

[15] Minear S (2010). Wnt proteins promote bone regeneration. Sci. Transl. Med..

[16] Glass DA (2005). Canonical Wnt signaling in differentiated osteoblasts controls osteoclast differentiation. Dev. Cell.

[17] Karner CM, Long F (2017). Wnt signaling and cellular metabolism in osteoblasts. Cell Mol. Life Sci..

[18] Bonewald LF, Johnson ML (2008). Osteocytes, mechanosensing and Wnt signaling. Bone.

[19] Weivoda MM (2016). Wnt Signaling Inhibits Osteoclast Differentiation by Activating Canonical and Noncanonical cAMP/PKA Pathways. J. Bone Min. Res.

[20] Robling AG (2008). Mechanical stimulation of bone in vivo reduces osteocyte expression of Sost/sclerostin. J. Biol. Chem..

[21] Westendorf JJ, Kahler RA, Schroeder TM (2004). Wnt signaling in osteoblasts and bone diseases. Gene.

[22] Janda CY, Waghray D, Levin AM, Thomas C, Garcia KC (2012). Structural basis of Wnt recognition by Frizzled. Science.

[23] Gurney A (2012). Wnt pathway inhibition via the targeting of Frizzled receptors results in decreased growth and tumorigenicity of human tumors. Proc. Natl Acad. Sci. USA.

[24] Bourhis E (2010). Reconstitution of a frizzled8.Wnt3a.LRP6 signaling complex reveals multiple Wnt and Dkk1 binding sites on LRP6. J. Biol. Chem..

[25] Ahn VE (2011). Structural basis of Wnt signaling inhibition by Dickkopf binding to LRP5/6. Dev. Cell.

[26] Janda CY (2017). Surrogate Wnt agonists that phenocopy canonical Wnt and beta-catenin signalling. Nature.

[27] Chen H (2020). Development of potent, selective surrogate WNT molecules and their application in defining frizzled requirements. Cell Chem. Biol..

[28] Zhao L, Li G, Chan KM, Wang Y, Tang PF (2009). Comparison of multipotent differentiation potentials of murine primary bone marrow stromal cells and mesenchymal stem cell line C3H10T1/2. Calcif. Tissue Int.

[29] Ryman JT, Meibohm B (2017). Pharmacokinetics of monoclonal antibodies. CPT Pharmacomet. Syst. Pharm..

[30] Day TF, Guo X, Garrett-Beal L, Yang Y (2005). Wnt/beta-catenin signaling in mesenchymal progenitors controls osteoblast and chondrocyte differentiation during vertebrate skeletogenesis. Dev. Cell.

[31] Bonnarens F, Einhorn TA (1984). Production of a standard closed fracture in laboratory animal bone. J. Orthop. Res..

[32] Kato M (2002). Cbfa1-independent decrease in osteoblast proliferation, osteopenia, and persistent embryonic eye vascularization in mice deficient in Lrp5, a Wnt coreceptor. J. Cell Biol..

[33] Hoang BH (2004). Expression of LDL receptor-related protein 5 (LRP5) as a novel marker for disease progression in high-grade osteosarcoma. Int J. Cancer.

[34] Li X (2005). Sclerostin binds to LRP5/6 and antagonizes canonical Wnt signaling. J. Biol. Chem..

[35] Lou S, Lv H, Li Z, Zhang L, Tang P (2018). Combination therapy of anabolic agents and bisphosphonates on bone mineral density in patients with osteoporosis: a meta-analysis of randomised controlled trials. BMJ Open.

[36] Cosman F (2014). Combination therapy for osteoporosis: a reappraisal. Bonekey Rep..

[37] Baron R, Kneissel M (2013). WNT signaling in bone homeostasis and disease: from human mutations to treatments. Nat. Med..

[38] Wei W (2011). Biphasic and dosage-dependent regulation of osteoclastogenesis by beta-catenin. Mol. Cell Biol..

[39] Chouinard L (2016). Carcinogenicity risk assessment of romosozumab: a review of scientific weight-of-evidence and findings in a rat lifetime pharmacology study. Regul. Toxicol. Pharm..

[40] Majidinia M, Sadeghpour A, Yousefi B (2018). The roles of signaling pathways in bone repair and regeneration. J. Cell Physiol..

[41] Zhong N, Gersch RP, Hadjiargyrou M (2006). Wnt signaling activation during bone regeneration and the role of Dishevelled in chondrocyte proliferation and differentiation. Bone.

[42] Chen Y (2007). Beta-catenin signaling plays a disparate role in different phases of fracture repair: implications for therapy to improve bone healing. PLoS Med..

[43] Morgan EF (2009). Micro-computed tomography assessment of fracture healing: relationships among callus structure, composition, and mechanical function. Bone.

[44] Lewiecki EM (2011). Sclerostin: a novel target for intervention in the treatment of osteoporosis. Disco. Med..

[45] Bouxsein ML (2010). Guidelines for assessment of bone microstructure in rodents using micro-computed tomography. J. Bone Min. Res..

[46] Turner CH, Burr DB (1993). Basic biomechanical measurements of bone: a tutorial. Bone.

