# Supplementary material for: Development of selective bispecific Wnt mimetics for bone loss and repair
Source: Nat Commun. 2021 May 31;12:3247. doi: 10.1038/s41467-021-23374-8 (PMC8167098; doi:10.1038/s41467-021-23374-8)
Supplement: Supplementary file 1 — Supplementary Information [file 41467_2021_23374_MOESM1_ESM.pdf]

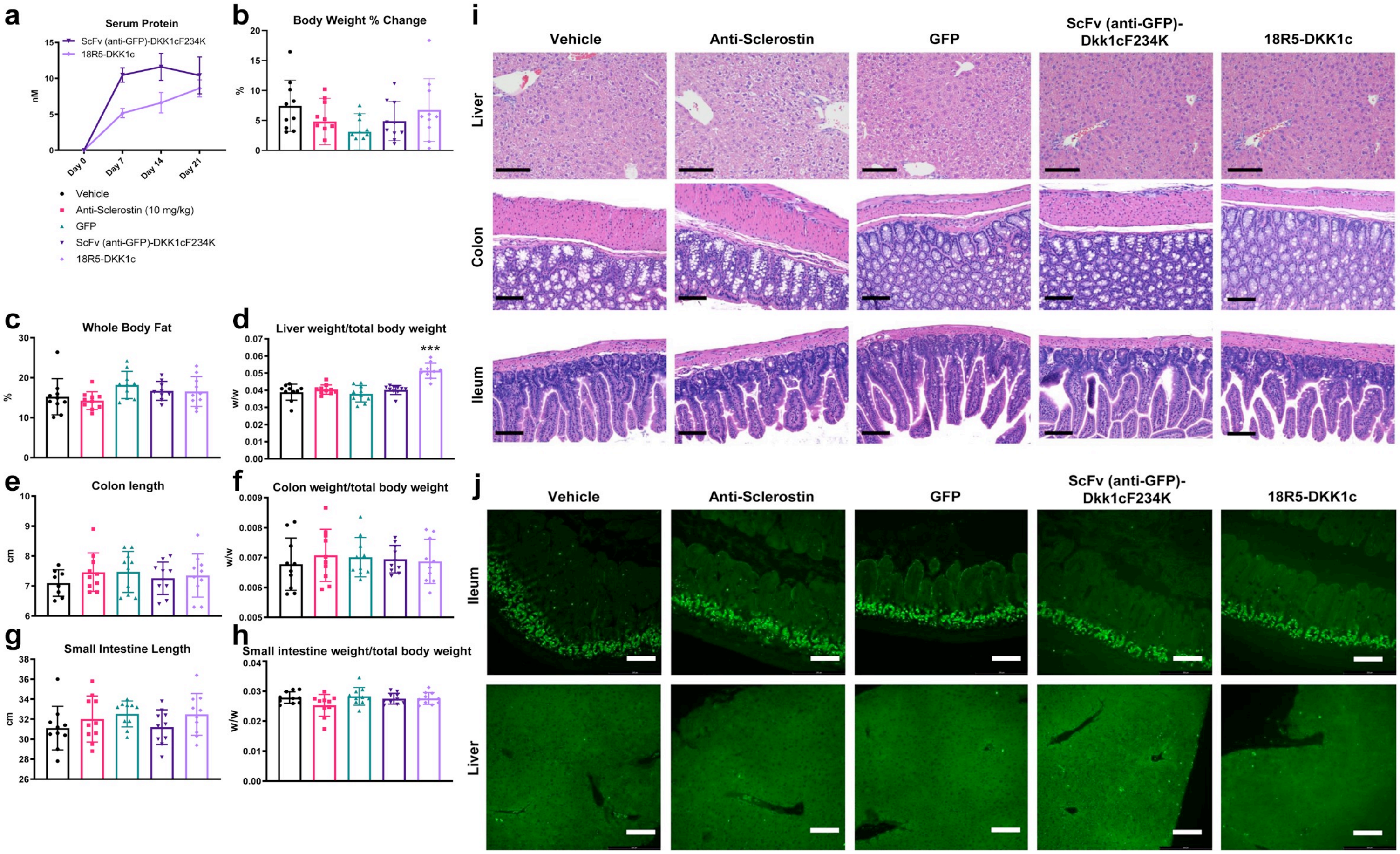

Supplementary Figure 1. Systemic mimetic expression causes no adverse effects over 28 days. Mice received a single tail vein injection of AAV containing DNA encoding various proteins (at a dose of $1x10^{11}$ viral particles/animal), or twice weekly x 4 weeks of anti-sclerostin antibody administered subcutaneously. Blood samples were collected at days 0, 7, 14, 21 and 28 of the treatment phase for both treated and control animals. (**a**) Flag-tagged proteins detected in the serum throughout the experiment, confirming systemic expression. (**b**) Infection over the 28-day period did not induce weight changes as measured at day 0 and at study termination on day 28 or (**c**) changes in whole body fat percentage as measured by dual-energy x-ray absorptiometry (DEXA) on day 28. At study termination, (**d**) whole liver, (**f**) colon, and (**h**) small intestine weights were individually normalized to whole body weight for all groups. Additionally, (**e**) colon and (**g**) small intestine lengths were measured for each animal. (**i**) For all animals, liver, colon, and small intestines were collected and processed for histopathology. Representative images (from n = 10 samples/group having similar results) of hematoxylin and eosin stained sections from each group are shown, where liver sections were made from caudate lobe for all groups and colon and ileum are sectioned longitudinally. Scale bar for all images, 50 um. Statistical significance was determined by one-way ANOVA, wherein part (d), vehicle vs. 18R5-Dkk1c, p=0.0002 (*** $p < 0.001$) (**j**) Small intestine and liver were collected and processed for Ki67 staining. Representative images (from n = 6 samples/group having similar results) are shown, and no differences were observed between groups. Scale bar for all images, 50 µm. (a-h) Graphs represent mean values ± SD, *n* = 10 mice/group.

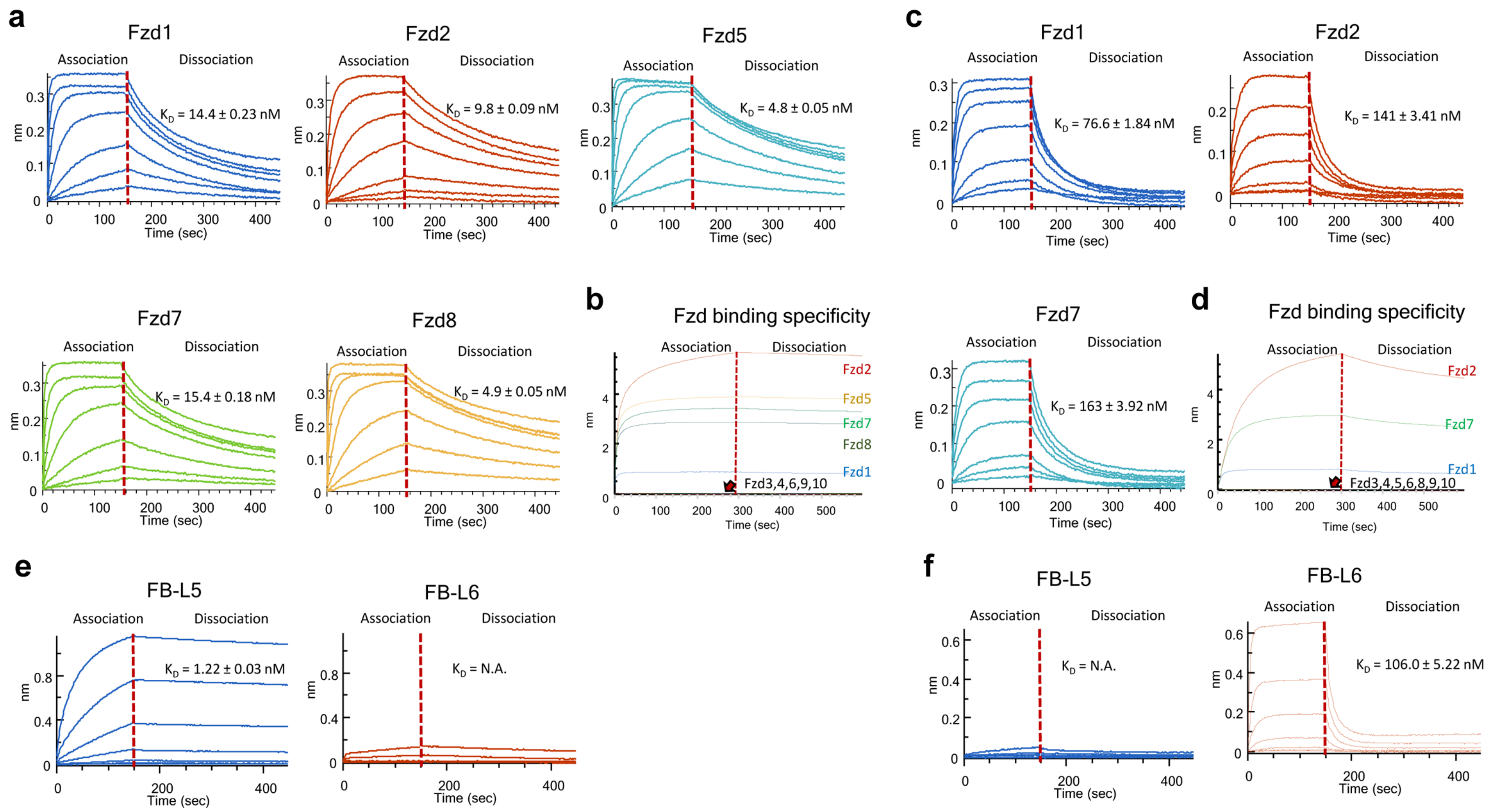

Supplementary Figure 2. The binding affinity and specificity profiles of FA, FB, L5, and L6. (**a**) The binding affinity of FA IgG to its target Fzd CRDs, $Fzd_{1,2,5,7,8}$ measured on Octet. (**b**) The binding specificity of FA IgG to total 10 Fzd CRDs were examined on Octet. (**c**) The binding affinity of FB IgG to its target Fzd CRDs, $FZD_{1,2,7}$ measured on Octet. (**d**) The binding specificity of FB IgG to total 10 Fzd CRDs were examined on Octet. (**e**) The binding affinities of FB-L5 (left) and FB-L6 (right) to Lrp5 measured on Octet. (**f**) The binding affinities of FB-L5 (left) and FB-L6 (right) to Lrp6E3E4 measured on Octet. N.A., not applicable.

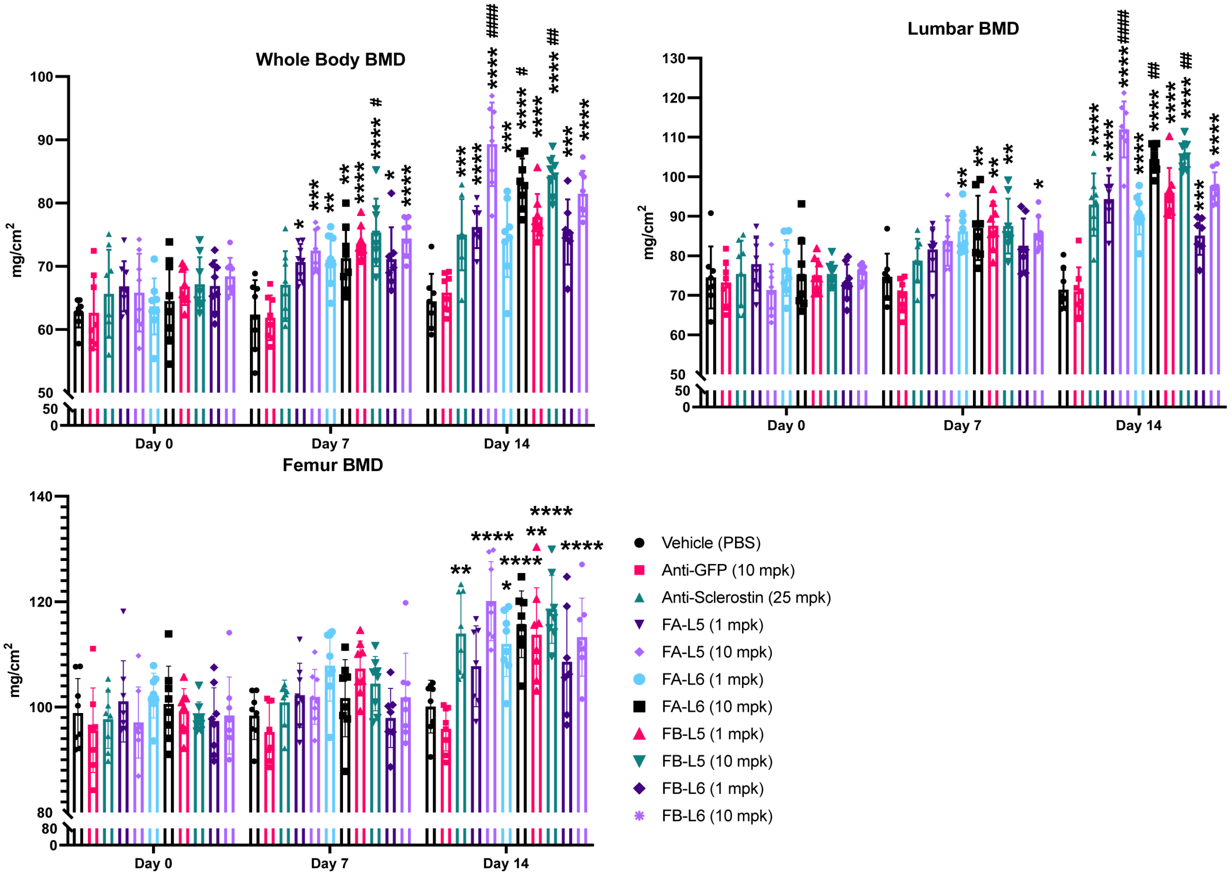

Supplementary Figure 3. In vivo DEXA characterization of Wnt mimetic molecules. 20-week old C57BL6/J female mice were injected bi-weekly for 14 days intraperitoneally with 1 and 10 mg/kg of FA-L6, FA-L5, FB-L6 and FB-L5 antibodies and compared to a negative control antibody (anti-GFP IgG 10 mg/kg), and a positive control (bi-weekly subcutaneous administration of 25 mg/kg anti-sclerostin antibody) and vehicle. Longitudinal DEXA measurements of whole-body BMD, lumbar (L4-L6) spine BMD and femur BMD quantified for days 0, 7, and 21. Graphs represent mean values ± SD, n = 8 mice/group. Statistical significance was determined by two-way ANOVA (Asterisks denote comparisons to Vehicle, hashtags denote comparisons to Anti-sclerostin. * p < 0.05, ** p < 0.01, *** p < 0.001, **** p < 0.0001. $^{\#}$ p < 0.05, $^{\#\#}$ p < 0.01, $^{\#\#\#\#}$ p < 0.0001), where the following letters define groups (a=Vehicle, b=Anti-sclerostin, c=FA-L5 (1 mpk), d=FA-L5 (10 mpk), e=FA-L6 (1 mpk), f=FA-L6 (10 mpk), g=FB-L5 (1 mpk), h=FB-L5 (10 mpk), i=FB-L6 (1 mpk), j=FB-L6 (10 mpk). Whole body BMD, Day 7, a vs. c, p=0.015, a vs. d, p=0.0007, a vs. e, p=0.008, a vs. f, p=0.006, a vs. g, p=<0.0001, a vs. h, p=<0.0001, a vs. i, p=0.0119, a vs. j, p=<0.0001, b vs. h, p=0.0157. Day 14, a vs. b, p=0.0004, a vs. c, p=<0.0001, a vs. d, p=<0.0001, a vs. e, p=0.001, a vs. f, p=<0.0001, a vs. g, p=<0.0001, a vs. h, p=<0.0001, a vs. i, p=0.0003, a vs. j, p=<0.0001, b vs. d, p=<0.0001, b vs. f, p=0.0181, b vs. h, p=0.0024. Lumbar BMD, Day 7, a vs. e, p=0.0072, a vs. f, p=0.0024, a vs. g, p=0.0012, a vs. h, p=0.0013, a vs. j, p=0.0118. Day 14, a vs. b, p=<0.0001, a vs c, p=<0.0001, a vs. d, p=<0.0001, a vs. e, p=<0.0001, a vs. f, p=<0.0001, a vs. g, p=<0.0001, a vs. h, p=<0.0001, a vs. i, p=0.0008, a vs. j, p=<0.0001, b vs. d, p=<0.0001, b vs. f, p=0.0067, b vs. h, p=0.0021. Femur BMD, Day 14, a vs. b, p=0.0014, a vs. d, p=<0.0001, a vs. e, p=0.0131, a vs. f, p=0.0001, a vs. g, p=0.0018, a vs. h, p=<0.0001, a vs. j, p=<0.0001.

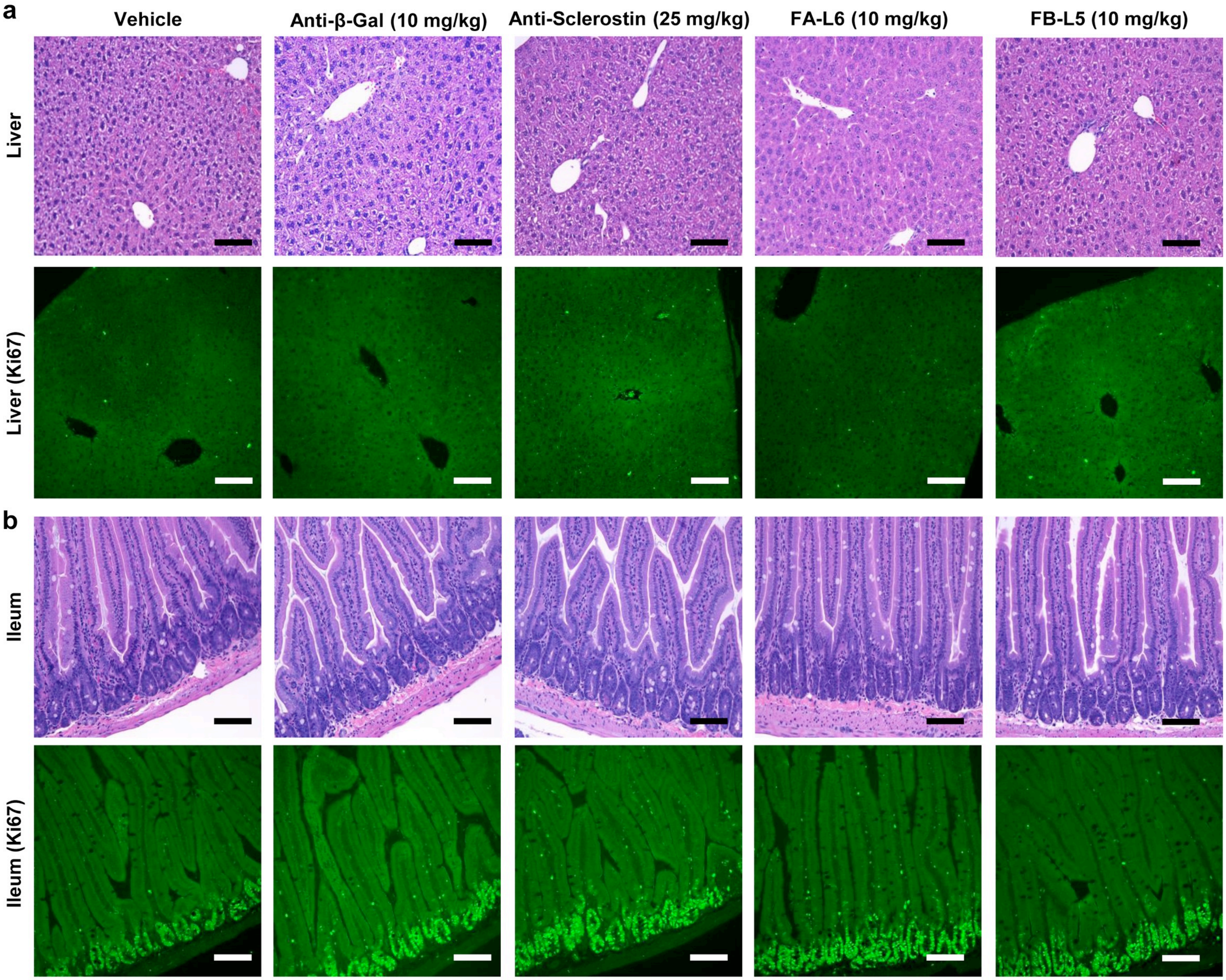

Supplementary Figure 4. Histology analysis of Wnt mimetic treated mice from Fig. 5F. (**a**) Liver and (**b**) intestine were collected and processed for histology and Ki67 staining. Representative images of hematoxylin and eosin, and Ki67 stained sections from each group are shown, no differences were observed between groups. Scale bar for all images, 50 µm. (a and b) Representative images from *n* = 6 samples/group having similar results.

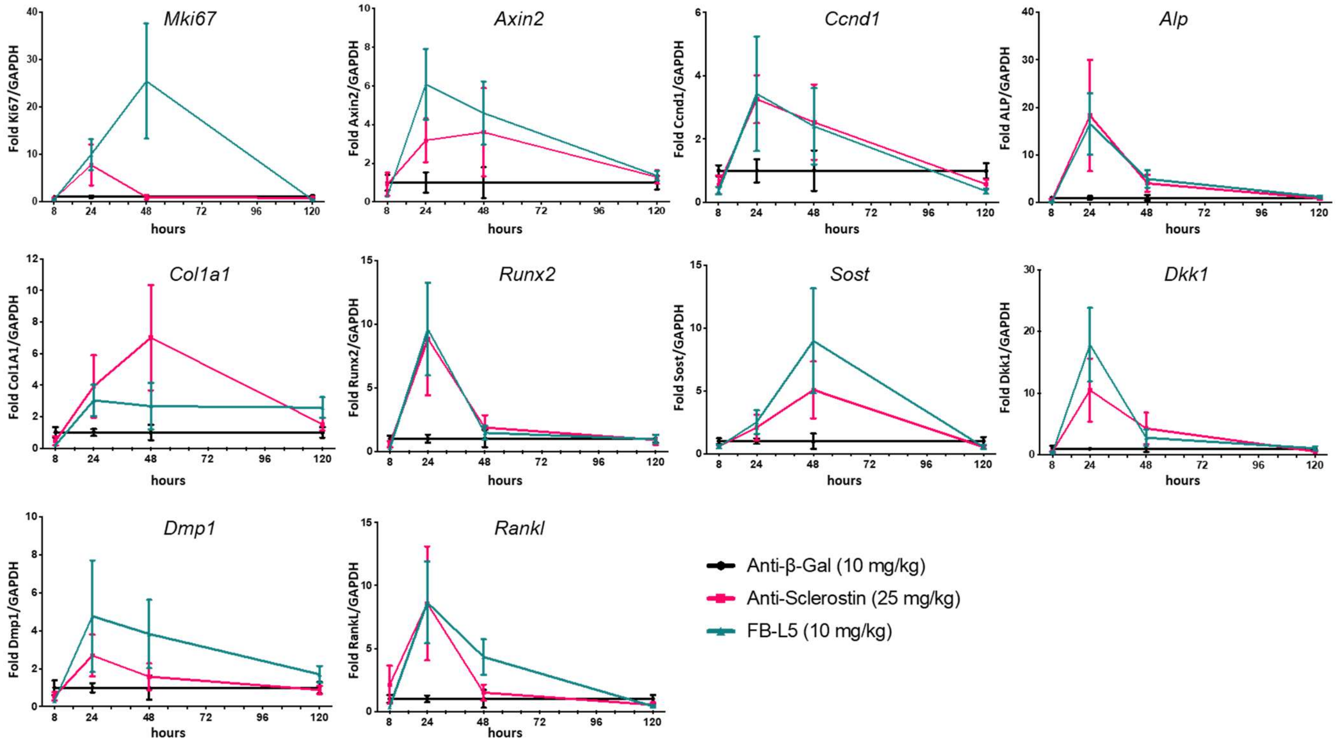

Supplementary Figure 5. qPCR on RNA isolated from bone after animals treated once and followed for various timepoints. Changes in gene expression in whole bone were measured over time to assess how FB-L5 regulates genetic markers related to proliferation and osteogenesis. C57BL6/J females, 12 weeks old (n=5 mice/group) were injected once intraperitoneally with anti-β-gal (10 mg/kg), FB-L5 (10 mg/kg), or once subcutaneously with anti-sclerostin antibody (25 mg/kg). Animals were euthanized at 24 and 48 hours after treatment, and tibias isolated for RNA extraction. RNA isolated from tibias was assayed for relative transcript levels of *Mki67*, *Axin2*, Cyclin D1 (*Ccnd1*), Alkaline phosphatase (*Alp*), Collagen Type 1 Alpha 1 chain (*Col1a1*), Runt Related Transcription Factor 2 (*Runx2*), sclerostin (*Sost*), Dickkopf Wnt signaling pathway inhibitor 1 (*Dkk1*), Dentin Matrix Acidic Phosphoprotein 1 (*Dmp1*), and Receptor activator of nuclear factor kappa-B ligand (*Rankl*). Graphs represent mean values ± SD.

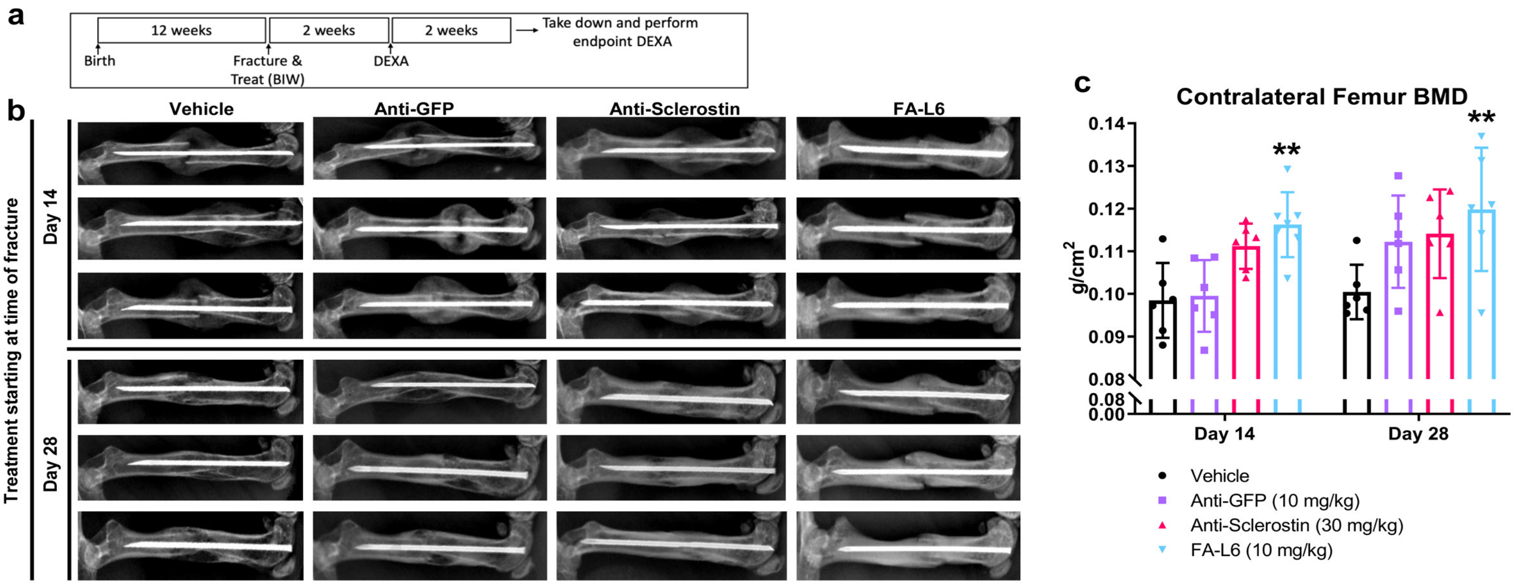

Supplementary Figure 6. Wnt mimetic antibodies increase bone accrual, but do not accelerate bone repair when given at the time of fracture. (**a**) Schematic of experimental design. 12-week old C57BL6/J female mice were surgically fractured adhering to the Einhorn fracture model. Mice were injected bi-weekly with intraperitoneal administration of vehicle, FA-L6 (10 mg/kg) antibody, negative control anti-GFP IgG (10 mg/kg) and bi-weekly subcutaneous administration of anti-sclerostin (30 mg/kg) antibody for 28 days. (**b**) Three representative x-ray images from each group at day 14 (top panel) and day 28 (bottom panel). (**c**) Longitudinal DEXA measurements were collected and non-fractured, contralateral femur BMD quantified for days 14 and 28. Statistical significance was determined by two-way ANOVA, Day 14, Vehicle vs. FA-L6, p=0.0088 and Day 28, Vehicle vs. FA-L6, p=0.0055.(c) Graphs represent mean values ± SD, ** $p < 0.01$, $n = 6$ mice/group.

Supplementary Table 1. Trabecular bone histomorphometric analysis at the metaphyseal region of proximal tibia. BV/TV, bone volume/total volume; GFP, green fluorescent protein; Tb.N, trabecular number; Tb.Th, trabecular thickness; Ob.S/BS, osteoblast surface/bone surface; Oc.S/BS, osteoclast surface/bone surface; O.Th, osteoid thickness; BS/BV, bone surface/bone volume ratio; Tb. Sp, trabecular separation; sLs/BS, Single label surface; OS/BS, osteoid surface as a percentage of bone surface; O.Ar, total osteoid area; OV/TV, osteoid volume as a percentage of tissue volume; OC.#/B.S, number of osteoclast/bone surface ratio.

**Parameter**	**Vehicle**	**Anti-Sclerostin**	**GFP**	**ScFv(anti-GFP)-DKK1cF234K**	**18R5-DKK1c**
BV/TV (%)	7.25 ± 1.8	10.6 ± 3.5	10.0 ± 2.6	9.58 ± 2.4	21.7 ± 3.0*
Tb.N (#/mm)	2.71 ± 0.5	3.29 ± 0.7	3.29 ± 0.6	3.04 ± 0.5	4.51 ± 0.4*
Tb.Th (μm)	26.6 ± 3.6	31.6 ± 4.8	29.9 ± 4.8	31.3 ± 4.5	48.1 ± 5.1*
Ob.S/BS (%)	3.06 ± 2.0	3.43 ± 0.9	5.47 ± 3.0	4.82 ± 2.1	7.12 ± 3.3*
Oc.S/BS (%)	6.45 ± 1.1	5.53 ± 0.9	5.37 ± 1.5	6.60 ± 1.7	4.83 ± 1.2
O.Th (μm)	1.82 ± 0.3	2.12 ± 0.5	2.28 ± 0.6	2.12 ± 0.5	2.90 ± 0.5*
BS/BV (mm/mm^2)	76.2 ± 1.8	64.6 ± 9.3	65.4 ± 8.3	68.8 ± 12.4	43.6 ± 6.3*
Tb. Sp (μm)	352.4 ± 66	288.8 ± 92	273.6 ± 49	317.1 ± 60	182.8 ± 31*
sLs/BS (%)	32.3 ± 11.6	17.4 ± 5.8*	30.7 ± 6.4	31.1 ± 6.8	32.3 ± 9.7
OS/BS (%)	7.72 ± 4.0	5.12 ± 3.8	15.75 ± 10.4	15.71 ± 5.9	16.96 ± 9.2*
O.Ar (mm^2)	0.001 ± 0.00	0.001 ± 0.00	0.003 ± 0.00	0.002 ± 0.00	0.004 ± 0.00*
OV/TV (%)	0.074 ± 0.05	0.069 ± 0.07	0.228 ± 0.20	0.189 ± 0.12	0.363 ± 0.2*
OC.#/B.S	3.41 ± 0.69	2.99 ± 0.26	2.88 ± 0.59	3.12 ± 0.75	2.59 ± 0.76

Data are expressed as mean ± SD. $n = 8$ mice/group. Histomorphometric analysis of proximal tibia confirming μCT volumetric readings, revealing systemic expression of 18R5-DKK1c resulted in significantly increased trabecular number, trabecular thickness, and osteoid thickness, wherein * indicates P value < 0.05 compared to vehicle control.

Supplementary Table 2. Cancellous and cortical microcomputed tomography analysis of distal and mid-diaphyseal femur. BV/TV, bone volume/total volume; Tb.N, trabecular number; Tb.Th, trabecular thickness; Tb. Sp, trabecular separation; Ct. Th, cortical thickness; Ct. Ar, cortical bone area; Tt.Ar, total cross-sectional area; Ct.Ar/At.Ar, cortical area fraction.

Parameter	Vehicle	Anti-Sclerostin	GFP	ScFv(anti-GFP)-DKK1cF234K	18R5-DKK1c
Trabecular					
BV/TV (%)	13.1 ± 4.7	19.5 ± 5.3*	13.1 ± 2.4	14.2 ± 4.4	29.8 ± 5.9*#
Tb.N (1/mm)	3.63 ± 0.84	4.74 ± 0.78*	3.85 ± 0.52	3.92 ± 0.71	5.71 ± 0.49*#
Tb.Th (mm)	0.036 ± 0.006	0.041 ± 0.004	0.034 ± 0.003	0.035 ± 0.005	0.052 ± 0.007*#
Tb. Sp (mm)	0.252 ± 0.061	0.175 ± 0.036*	0.231 ± 0.039	0.228 ± 0.055	0.125 ± 0.024*
Mean/Density (mg HA/ccm)	760.4 ± 12.6	761.6 ± 14.1	760.3 ± 8.2	757.6 ± 16.4	790.3 ± 17.2*
Cortical					
Ct. Th. (mm)	0.172 ± 0.011	0.213 ± 0.019*	0.173 ± 0.007	0.171 ± 0.012	0.203 ± 0.019*
Ct.Ar (mm^2)	0.812 ± 0.055	1.063 ± 0.122*	0.79 ± 0.061	0.836 ± 0.066	0.97 ± 0.101*
Tt.Ar (mm^2)	0.84 ± 0.054	1.092 ± 0.123*	0.816 ± 0.061	0.864 ± 0.067	0.999 ± 0.102*
Ct.Ar./Tt.Ar	0.967 ± 0.004	0.973 ± 0.002*	0.968 ± 0.003	0.968 ± 0.002	0.97 ± 0.007

Data are expressed as mean value ± SD relative to vehicle. $n = 8$/group. *$P < 0.05$ compared to vehicle treated group, # $P < 0.05$ compared to anti-sclerostin treated group.

Supplementary Table 3. Changes in gene expression in bone with Wnt surrogate molecule and anti-sclerostin therapy in mice. Anti-β-Gal (anti-β-galactosidase); *Col1a1*, type 1 alpha 1 chain; *Dmp1*, dentin matrix acidic phosphoprotein 1; *Alp*, alkaline phosphatase; *Rankl*, receptor activator of nuclear kappa-B ligand; *Dkk1*, Dickkopf Wnt signaling pathway inhibitor 1; *Sost*, sclerostin; *Ccnd1*, cyclin D1.

		**Treatment**		
**Gene**	**Time (hr)**	**Anti-β-Gal 10 mg/kg**	**Anti-Sclerostin 25 mg/kg**	**FB-L5 10 mg/kg**
Runx2	24	1.0 ± 0.3	8.8 ± 4.4*	9.6 ± 3.6*
	48	1.0 ± 0.7	1.9 ± 1.0	1.5 ± 0.5
Col1a1	24	1.0 ± 0.2	3.9 ± 2.0*	3.0 ± 1.0
	48	1.0 ± 0.5	7.0 ± 3.3	2.7 ± 1.5
Dmp1	24	1.0 ± 0.2	2.7 ± 1.1	4.8 ± 2.9
	48	1.0 ± 0.6	1.6 ± 0.7	3.8 ± 1.8
Alp	24	1.0 ± 0.4	18.3 ± 11.7*	16.5 ± 6.4*
	48	1.0 ± 0.6	4.0 ± 1.8	5.0 ± 1.9
Rankl	24	1.0 ± 0.3	8.6 ± 4.5*	8.7 ± 3.3*
	48	1.0 ± 0.7	1.5 ± 0.6	4.3 ± 1.4
Dkk1	24	1.0 ± 0.2	10.5 ± 5.1	17.9 ± 6.0*
	48	1.0 ± 0.5	4.3 ± 2.5	2.8 ± 1.3
Sost	24	1.0 ± 0.2	2.1 ± 1.0	2.5 ± 0.9*
	48	1.0 ± 0.6	5.1 ± 2.3	9.0 ± 4.2
Ccnd1	24	1.0 ± 0.4	3.3 ± 0.8	3.4 ± 1.8
	48	1.0 ± 0.6	2.5 ± 1.2	2.4 ± 1.2
Axin2	24	1.0 ± 0.5	3.2 ± 1.1	6.1 ± 1.8*
	48	1.0 ± 0.8	3.6 ± 2.3	4.6 ± 1.6*
Mki67	24	1.0 ± 0.3	7.7 ± 4.3*	10.0 ± 3.2*
	48	1.0 ± 0.5	1.0 ± 0.5	25.5 ± 12.2*

Data are expressed as mean value ± SE for fold induction relative to Anti-β-Gal. $n = 5$/group. *$P < 0.05$ compared to Anti-β-Gal-treated group.

Supplementary Table 4. List of all primers used.

Product Number	Item description	Primer Set Information
4331182	Mouse Fzd1 taqman probe/primer set Mm00445405_s1	Sequence not disclosed by vendor
4331182	Mouse Fzd 2 taqman probe/primer set Mm02524776_s1	Sequence not disclosed by vendor
4331182	Mouse Fzd 3 taqman probe/primer set Mm00445423_m1	Sequence not disclosed by vendor
4331182	Mouse Fzd 4 taqman probe/primer set Mm00433382_m1	Sequence not disclosed by vendor
4331182	Mouse Fzd 5 taqman probe/primer set Mm00445623_s1	Sequence not disclosed by vendor
4331182	Mouse Fzd 6 taqman probe/primer set Mm00433387_m1	Sequence not disclosed by vendor
4331182	Mouse Fzd 7 taqman probe/primer set Mm00433409_s1	Sequence not disclosed by vendor
4331182	Mouse Fzd 8 taqman probe/primer set Mm01234717_s1	Sequence not disclosed by vendor
4331182	Mouse Fzd 9 taqman probe/primer set Mm01206511_s1	Sequence not disclosed by vendor
4331182	Mouse Fzd 10 taqman probe/primer set Mm00558396_s1	Sequence not disclosed by vendor
4331182	Mouse Lrp5 taqman probe/primer set Mm01227476_m1	Sequence not disclosed by vendor
4331182	Mouse Lrp6 taqman probe/primer set Mm00999795_m1	Sequence not disclosed by vendor
4331182	Mouse ActB taqman probe/primer set Mm02619580_g1	Sequence not disclosed by vendor
4331182	Mouse Ccnd1 taqman probe/primer set Mm00432359_m1	Sequence not disclosed by vendor
4331182	Mouse Axin2 taqman probe/primer set Mm00443610_m1	Sequence not disclosed by vendor
4331182	Mouse Ki67 taqman probe/primer set Mm01278617_m1	Sequence not disclosed by vendor
4331182	Mouse Col1a1 taqman probe/primer set Mm00801666_g1	Sequence not disclosed by vendor
4331182	Mouse Alpl taqman probe/primer set Mm00475834_m1	Sequence not disclosed by vendor
4331182	Mouse Runx2 taqman probe/primer set Mm00501584_m1	Sequence not disclosed by vendor
4331182	Mouse Dmp1 taqman probe/primer set Mm01208363_m1	Sequence not disclosed by vendor
4331182	Mouse SOST taqman probe/primer set Mm00470479_m1	Sequence not disclosed by vendor
4331182	Mouse Dkk1 taqman probe/primer set Mm00438422_m1	Sequence not disclosed by vendor
4331182	Mouse RANKL taqman probe/primer set Mm00441906_m1	Sequence not disclosed by vendor
4331182	Human Fzd1 taqman probe/primer set	Sequence not disclosed by

	Hs00268943_s1	vendor
4331182	Human Fzd2 taqman probe/primer set Hs00361432_s1	Sequence not disclosed by vendor
4331182	Human Fzd3 taqman probe/primer set Hs00907280_m1 / Hs00184043_m1	Sequence not disclosed by vendor
4331182	Human Fzd4 taqman probe/primer set Hs00201853_m1	Sequence not disclosed by vendor
4331182	Human Fzd5 taqman probe/primer set Hs00258278_s1	Sequence not disclosed by vendor
4331182	Human Fzd6 taqman probe/primer set Hs01095627_m1 / Hs00171574_m1	Sequence not disclosed by vendor
4331182	Human Fzd7 taqman probe/primer set Hs00275833_s1	Sequence not disclosed by vendor
4331182	Human Fzd8 taqman probe/primer set Hs00259040_s1	Sequence not disclosed by vendor
4331182	Human Fzd9 taqman probe/primer set Hs00268954_s1	Sequence not disclosed by vendor
4331182	Human Fzd10 taqman probe/primer set Hs00273077_s1	Sequence not disclosed by vendor
4331182	Human Lrp5 taqman probe/primer set Hs00182031_m1	Sequence not disclosed by vendor
4331182	Human Lrp6 taqman probe/primer set Hs00233945_m1	Sequence not disclosed by vendor
4331182	Human ACTB taqman probe/primer set Hs01060665_g1	Sequence not disclosed by vendor
