# Supplementary material for: Development of selective bispecific Wnt mimetics for bone loss and repair
Source: Nat Commun. 2021 May 31;12:3247. doi: 10.1038/s41467-021-23374-8 (PMC8167098; doi:10.1038/s41467-021-23374-8)
Supplement: Supplementary file 2 — Reporting Summary [file 41467_2021_23374_MOESM2_ESM.pdf]

nature research

Corresponding author(s): Wen-Chen Yeh and Yang Li

Last updated by author(s): 01/20/2021

Reporting Summary

Nature Research wishes to improve the reproducibility of the work that we publish. This form provides structure for consistency and transparency in reporting. For further information on Nature Research policies, see our Editorial Policies and the Editorial Policy Checklist.

Statistics

For all statistical analyses, confirm that the following items are present in the figure legend, table legend, main text, or Methods section.

n/a	Confirmed	
☐	☒	The exact sample size (n) for each experimental group/condition, given as a discrete number and unit of measurement
☐	☒	A statement on whether measurements were taken from distinct samples or whether the same sample was measured repeatedly
☐	☒	The statistical test(s) used AND whether they are one- or two-sided<br>*Only common tests should be described solely by name; describe more complex techniques in the Methods section.*
☒	☐	A description of all covariates tested
☒	☐	A description of any assumptions or corrections, such as tests of normality and adjustment for multiple comparisons
☐	☒	A full description of the statistical parameters including central tendency (e.g. means) or other basic estimates (e.g. regression coefficient) AND variation (e.g. standard deviation) or associated estimates of uncertainty (e.g. confidence intervals)
☐	☒	For null hypothesis testing, the test statistic (e.g. F, t, r) with confidence intervals, effect sizes, degrees of freedom and P value noted<br>*Give P values as exact values whenever suitable.*
☒	☐	For Bayesian analysis, information on the choice of priors and Markov chain Monte Carlo settings
☒	☐	For hierarchical and complex designs, identification of the appropriate level for tests and full reporting of outcomes
☒	☐	Estimates of effect sizes (e.g. Cohen's d, Pearson's r), indicating how they were calculated

Our web collection on statistics for biologists contains articles on many of the points above.

Software and code

Policy information about availability of computer code

Data collection	No software was used
Data analysis	Prism

For manuscripts utilizing custom algorithms or software that are central to the research but not yet described in published literature, software must be made available to editors and reviewers. We strongly encourage code deposition in a community repository (e.g. GitHub). See the Nature Research guidelines for submitting code & software for further information.

Data

Policy information about availability of data

All manuscripts must include a data availability statement. This statement should provide the following information, where applicable:

- Accession codes, unique identifiers, or web links for publicly available datasets
- A list of figures that have associated raw data
- A description of any restrictions on data availability

The authors declare that the data supporting the findings of this study are available within the paper and its supplementary figures.

Field-specific reporting

Life sciences study design

All studies must disclose on these points even when the disclosure is negative.

Sample size	Sample size selection is based upon power calculations, preliminary data and past experience. A G*Power 3.1 analysis indicates that 8 mice/group/experiment are required to observe, among many different outcomes, a 3-fold induction of gene expression with 99% power and a significance of p<0.05.
Data exclusions	No data were excluded from analyses in any of the presented data
Replication	Biological replicates are indicated within each experiment and documented within the data graphs or tables.
Randomization	Animals were randomized according to weight, or in the case of ovariectomy, animals were randomized according to bone mineral density as measured by DEXA. For all other studies using cell-lines, no randomization was needed and proper plating controls were included for consistency. No other randomization processes were used for other analyses.
Blinding	All analyses for animal related outcomes were blinded. Histological analyses were blinded to the histologist scoring. For affinity measurements, gene expression analysis, and STF activity, no blinding was deemed necessary.

Reporting for specific materials, systems and methods

We require information from authors about some types of materials, experimental systems and methods used in many studies. Here, indicate whether each material, system or method listed is relevant to your study. If you are not sure if a list item applies to your research, read the appropriate section before selecting a response.

Materials & experimental systems

n/a	Involved in the study	
☐	☒	Antibodies
☐	☒	Eukaryotic cell lines
☒	☐	Palaeontology and archaeology
☐	☒	Animals and other organisms
☒	☐	Human research participants
☒	☐	Clinical data
☒	☐	Dual use research of concern

Methods

n/a	Involved in the study	
☒	☐	ChIP-seq
☒	☐	Flow cytometry
☒	☐	MRI-based neuroimaging

Antibodies

Antibodies used

AffiniPure Goat Anti-Human IgG, Fcγ Fragment Specific (min X Bov,Ms,Rb Sr Prot) antibody(Jackson ImmunoResearch Labs Cat# 109-005-170, RRID:AB_2810885)

AffiniPure Donkey Anti-Human IgG, Fc_ Fragment Specific (min X Bov,Hrs,Ms Sr Prot) antibody(Jackson ImmunoResearch Labs Cat# 709-005-098, RRID:AB_2340482)

Ki-67 Monoclonal Antibody (SolA15), eBioscience™ from Thermo Fisher Scientific, catalog # 14-5698-82, RRID AB_10854564.

Anti-Flag antibody from R&D Systems HAM85291: DYKDDDDK Epitope Tag, HRP, Clone 1042E

Note: The anti-sclerostin antibody used in our experiments was prepared in-house based on published sequence data (44)

Validation

Below information provided from data sheets on vendor website:

AffiniPure Goat Anti-Human IgG, Fcγ Fragment Specific (min X Bov,Ms,Rb Sr Prot) antibody: Based on immunoelectrophoresis and/or ELISA, the antibody reacts with the Fc portion of human IgG heavy chain but not with the Fab portion of human IgG. No antibody was detected against human IgM or IgA, or against non-immunoglobulin serum proteins. The antibody has been tested by ELISA and/or solid-phase adsorbed to ensure minimal cross-reaction with bovine, mouse and rabbit serum proteins, but it may cross-react with immunoglobulins from other species.

AffiniPure Donkey Anti-Human IgG, Fc_ Fragment Specific (min X Bov,Hrs,Ms Sr Prot) antibody: Based on immunoelectrophoresis and/or ELISA, the antibody reacts with the Fc portion of human IgG heavy chain but not with the Fab portion of human IgG. No antibody was detected against human IgM or IgA, or against non-immunoglobulin serum proteins. The antibody has been tested by ELISA and/or solid-phase adsorbed to ensure minimal cross-reaction with bovine, horse and mouse serum proteins, but it may cross-react with immunoglobulins from other species.

Ki-67 Monoclonal Antibody (SolA15): This Antibody was verified by Cell treatment to ensure that the antibody binds to the antigen stated.

Eukaryotic cell lines

Policy information about cell lines

Cell line source(s)	HEK293 STF: ATCC Cat# CRL-3249, RRID:CVCL_AQ26. C3H10T1/2: ATCC Cat# CCL-226, RRID:CVCL_0190. MC3T3: ATCC Cat# CRL-2593, RRID:CVCL_5440
Authentication	None of the cell lines used were authenticated.
Mycoplasma contamination	Cell lines have not been tested for mycoplasma contamination.
Commonly misidentified lines (See ICLAC register)	None

Animals and other organisms

Policy information about studies involving animals; ARRIVE guidelines recommended for reporting animal research

Laboratory animals	For all studies, female mice were used. For each study, the age of the mice is noted, ranging from 4 weeks to 1 year old at the beginning of a study. On arrival, animals were randomly assigned to group (5 animals/cage) housing and provided with rodent diet and water ad libitum. All mice were maintained on a 12:12-hour light/dark photoperiod at an ambient temperature of 22 +/- 2 °C. All animal studies utilized C57BL/6 mice obtained from The Jackson Laboratory (JAX stock #000664)
Wild animals	The study did not use any wild animals.
Field-collected samples	The study did not use samples collected from the field.
Ethics oversight	All animal experiments were performed according to national ethical guidelines in addition to the guidance and approval by the Institutional Animal Care and Use Committee (IACUC) of Surrozen, Inc.

Note that full information on the approval of the study protocol must also be provided in the manuscript.
